# Supplementary material for: Evolution of acoustic communication in blind cavefish
Source: Nat Commun. 2019 Sep 17;10:4231. doi: 10.1038/s41467-019-12078-9 (PMC6748933; doi:10.1038/s41467-019-12078-9)
Supplement: Supplementary file 1 — Supplementary Information [file 41467_2019_12078_MOESM1_ESM.pdf]

Supplementary Information

Evolution of acoustic communication in blind cavefish

Hyacinthe et al.

Contains:

5 Supplementary figures, 3 Supplementary tables, and 1 Supplementary reference.

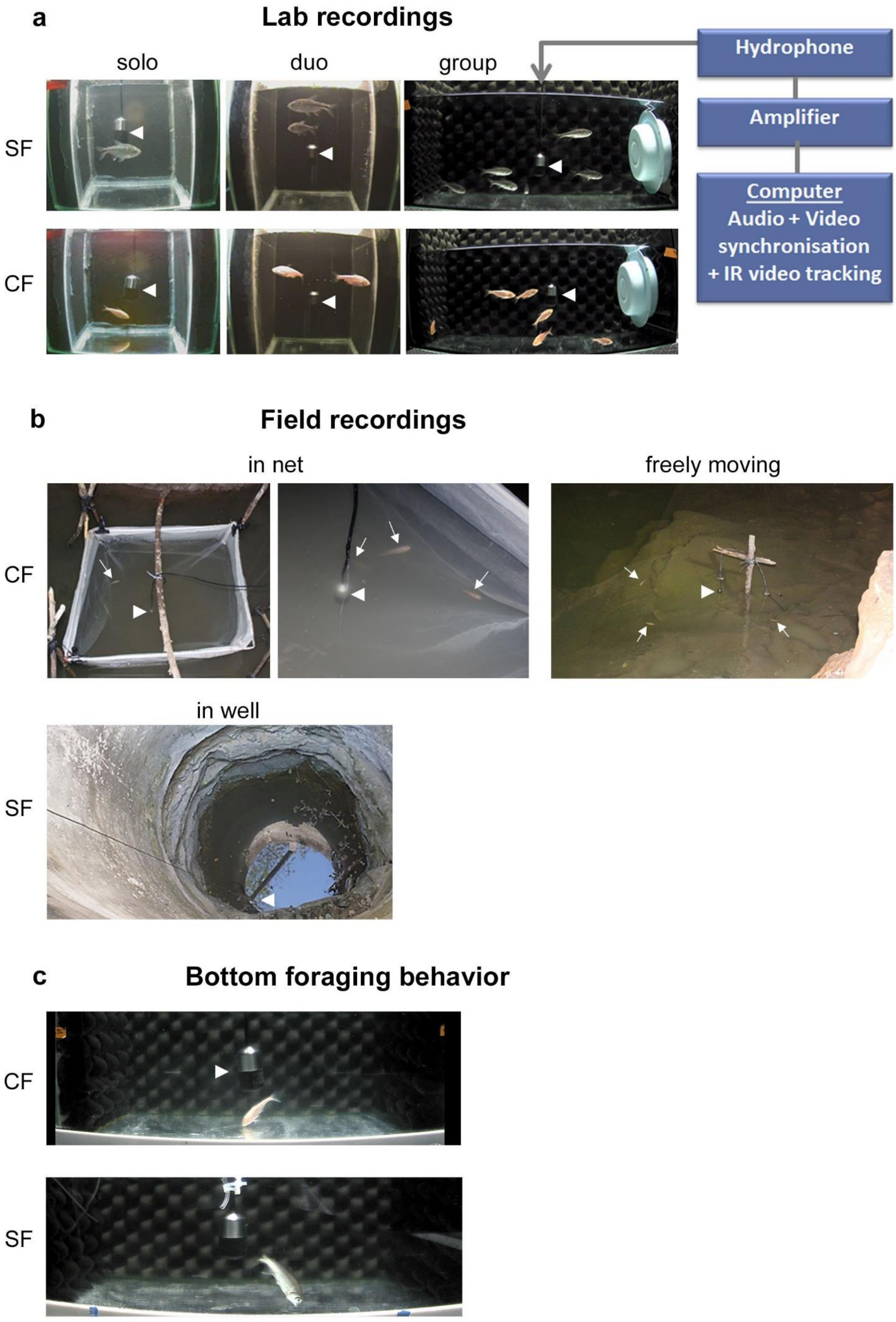

Supplementary Figure 1: experimental setups for acoustic recordings, in the lab and in the field.

In all pictures, the white arrowhead indicates the hydrophone, and white arrows point to fish.

a, In the lab, differently-sized tanks covered with soundproof foam except on one side were used to perform solo, duo, or group acoustic recordings in Pachón cavefish (CF) or surface fish

(SF). The hydrophone was placed in the middle of the tank. In large tanks (right), a loudspeaker (light blue) was used to perform play-backs.

b, In the field. In caves, recordings were performed on groups of 10-12 cavefish placed in a 1m x 1m square net, installed in their natural pool, with the hydrophone placed at the center (left photos in the Pachón cave). Alternatively, recordings could also be obtained from freely swimming fish (right photo, in the Sabinos cave) or in plastic pools (not shown). For surface fish, recordings were obtained in a well with still, non-running water favorable for acoustic recordings (bottom photo). Hundreds of surface fish were swimming in this well, as verified after climbing down the well.

c, Typical cavefish foraging behavior induced by perfusion of a food odor. Upon chemosensory stimulation, Pachón cavefish adopt a typical foraging posture, with the body making an approximate 45° angle to the substrate. This behavior was described by Schemmel in 1980 [1] and confers them a high efficiency to find and absorb food. Surface fish can also show this behavior, although with a more vertical position and to a lesser extent.

All pictures are extracted from videos or were taken by authors (SR) during field work.

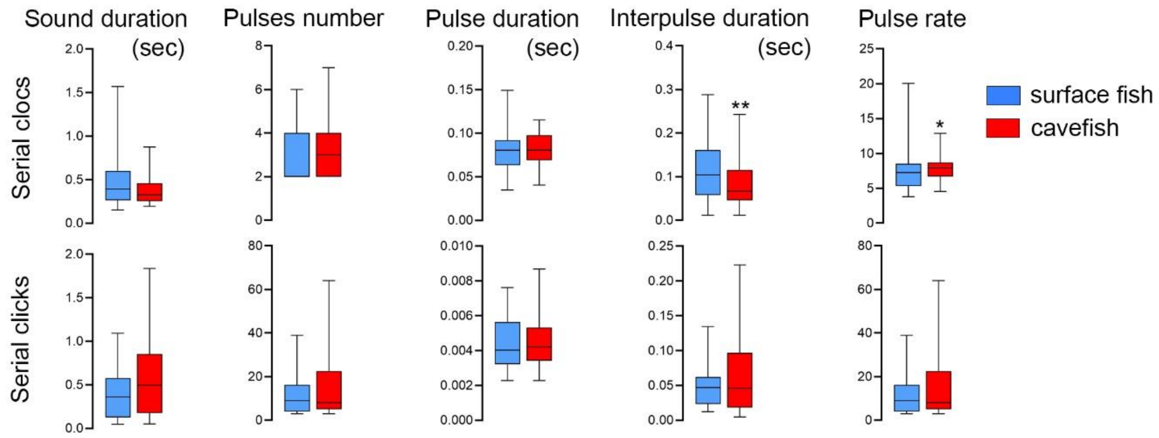

Supplementary Figure 2: comparison of temporal frequency parameters between surface fish and cavefish, for Serial Clicks and Serial Clocs.

Comparisons of 5 parameters (total sounds duration, pulse number per sound, mean pulse duration, mean inter-pulse duration, pulse rate) in the two *Astyanax* morphs (SF in blue, Pachón CF in red). Mann Whitney tests: Serial Clocs interpulse duration and pulse rate U = 1451 and U = 1506, respectively (*p<0.05, **p<0.01). See also Supplementary Data 3 for exhaustive statistics. Source data are provided as a Source Data file.

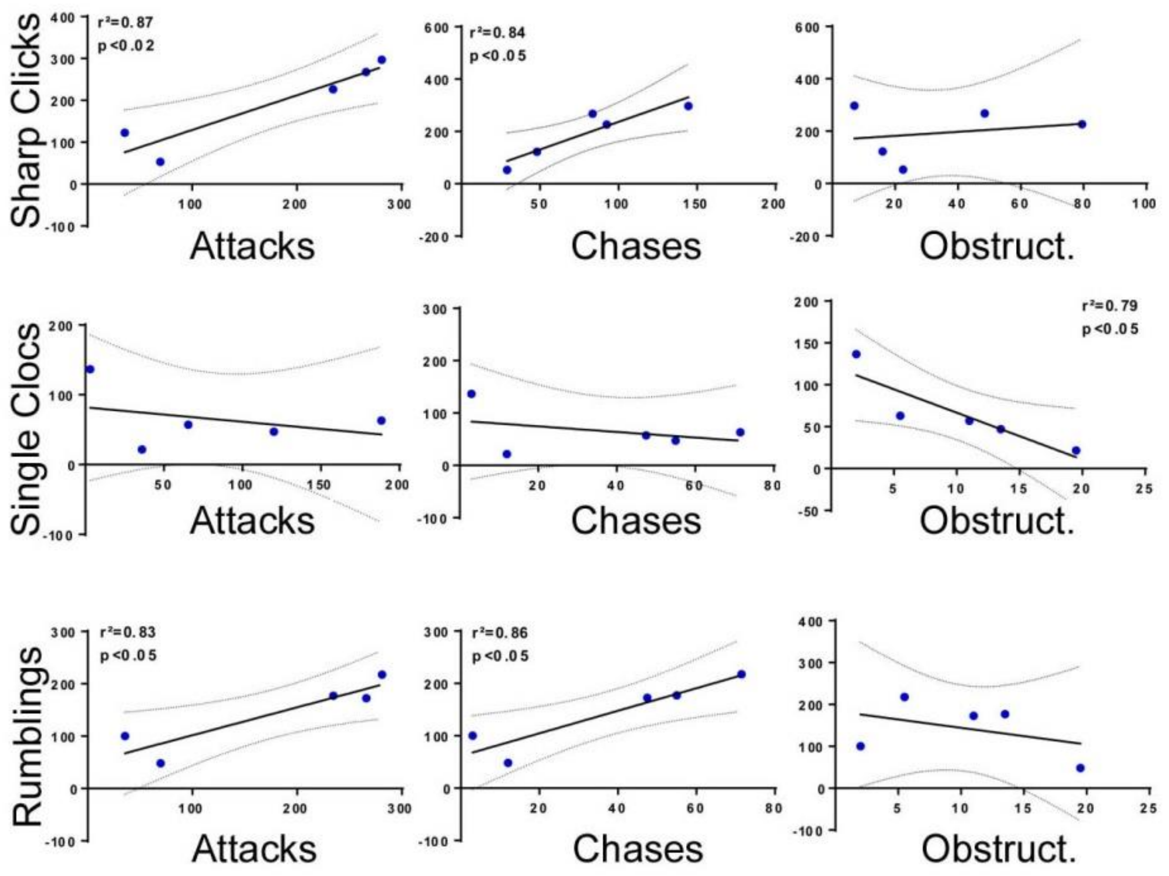

Supplementary Figure 3: relationships between sound production and agonistic behaviors in the resident-intruder assay in surface fish.

Linear regressions showing the relationships, in SF, between behaviors (attacks, chases, or obstructions) and sounds (Sharp Clicks, Clocs, or Rumblings) produced. Y-axis is the number of sounds produced and X-axis is the number of agonistic events observed. Two-way ANOVA-RM F (Sharp clicks *versus* attacks, chases, obstructions: 20.82, 16.02, 0.15); F (Single clocs *versus* attacks, chases, obstructions: 0.53, 0.05, 0.09); F (Rumblings *versus* attacks, chases, obstructions: 14.47, 30.29, 0.17). Source data are provided as a Source Data file.

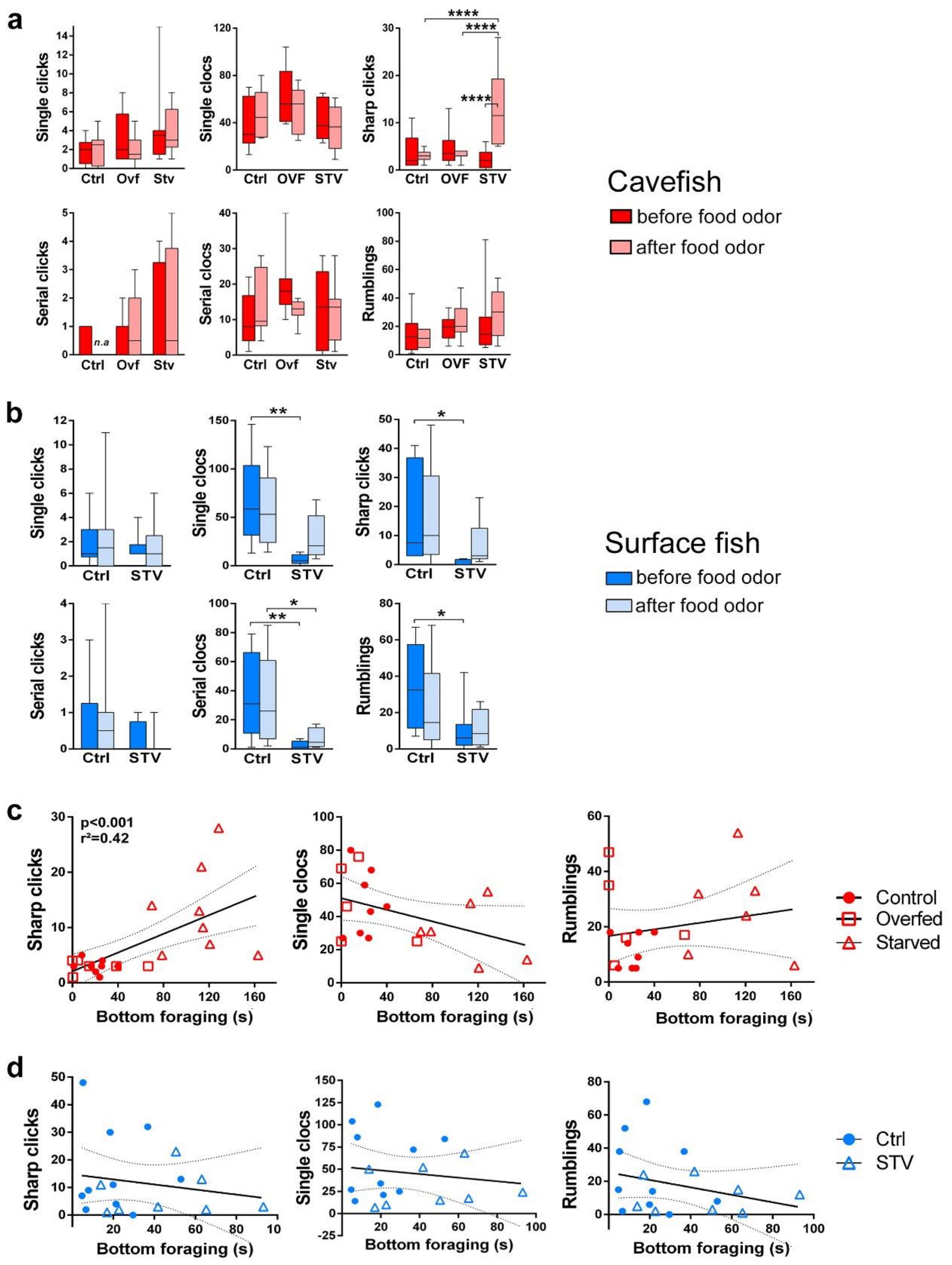

Supplementary Figure 4: relationships between sound production and bottom feeding behavior in cavefish and surface fish.

a, b, Sound production in control (CTRL), starved (STV) or overfed (OVF) cavefish (a) and surface fish (b) before (dark color boxes) and after (light color boxes) the perfusion of a chemosensory stimulus. Two-way ANOVA-RM (a, interactions: $F_{(2,21)} = 13.32$; b, interactions: $F_{(1,16)} = 2.5$; p=0.13 (Single clocs), $F_{(1,16)} = 2.01$; p=0.176 (Sharp clicks), $F_{(1,16)} = 1.27$; p=0.27

(Serial clocs), $F_{(1,16)}$ =1.36; p=0.26). **c, d,** Relationships between sound production and bottom foraging behavior in cavefish (red, c) and surface fish (blue, d). X-axis is the number of sounds produced and Y-axis is the time spent in foraging. When significant, the correlation coefficient is indicated. Full circles correspond to control fish, triangles correspond to starved fish, squares correspond to overfed fish (see also Methods). See also Supplementary Data 3 for exhaustive statistics. Source data are provided as a Source Data file.

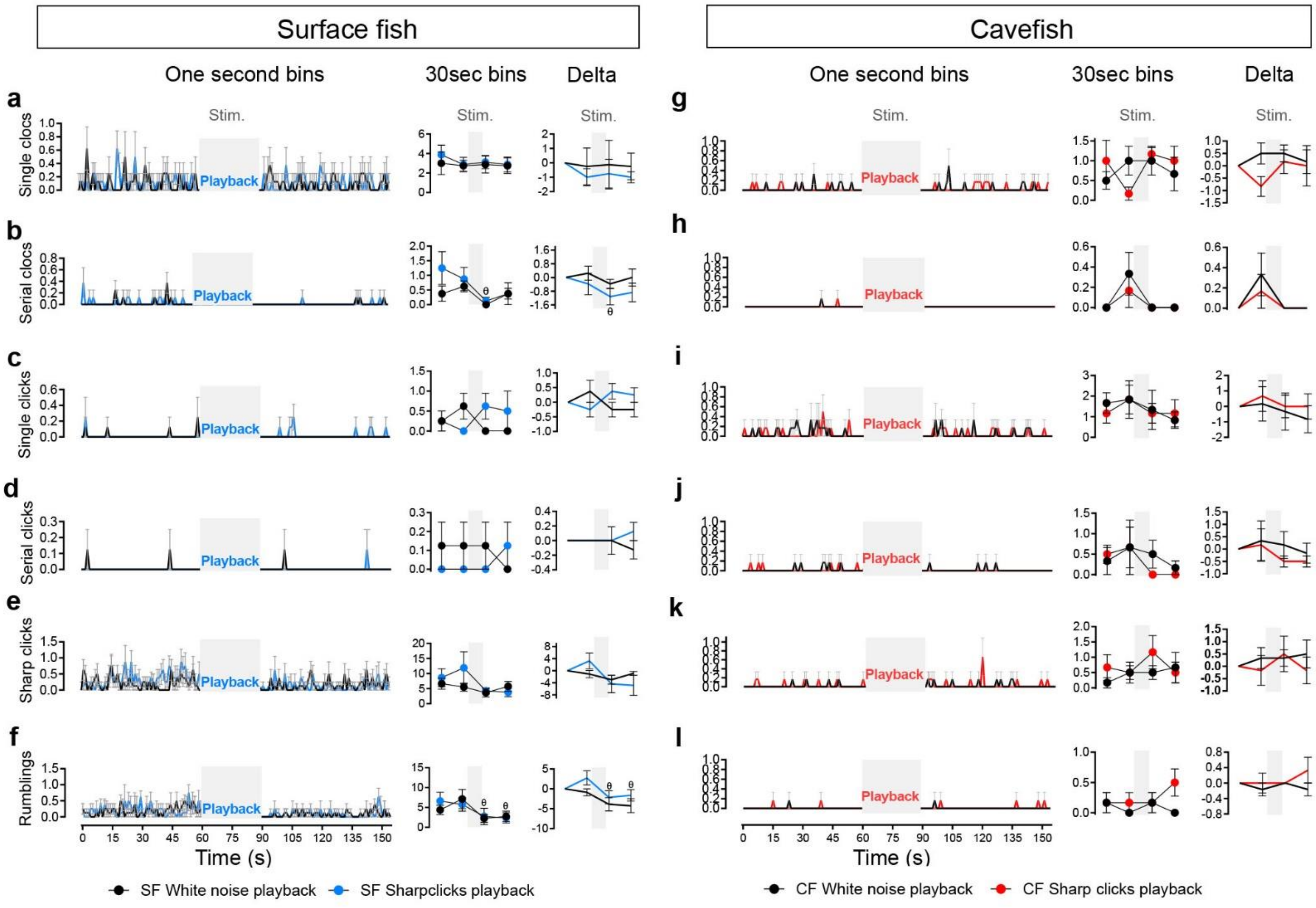

Supplementary Figure 5: sound production after Sharp Clicks or white noise play-backs, in cavefish and surface fish.

Sound production was measured before and after play-back (grey area, Stim) of Sharp Clicks (colored lines) or white noise (black lines) in surface fish (blue graphs, left columns) and cavefish (red graphs, right columns). Results are presented with one second bins and 30 seconds bins (first and second graph of each panel), as well as the variation respective to the "before sound play-back" condition (third graph of each panel shows Delta, i.e., difference to the first 30 seconds "before sound play-back" condition).

a,g, Single Clocs. **b,h,** Serial Clocs. **c,j,** Single Clicks. **d,j,** Serial Clicks. **e,k,** Sharp Clicks. **f,l,** Rumblings. Data are presented as means (± SEM); Two-way ANOVA-RM, interactions: $F_{(4,42)}$ =0.84; p=0.48 (b), $F_{(3,42)}$ =1.00; p=0.40 (f); Bonferroni *posthocs*: p values <0.05-0.0001 between time points are indicated as compared to baseline before stimulation (θ). See also Supplementary Data 3 for exhaustive statistics.

Supplementary Table 1: summary statistics for PCAs and pFDA analyses shown in Figure 1 and 2.

A. PCA analysis on simple sounds acoustic parameters (Fig. 1g).

	Variance	% of variance	Cumulative % of variance	SD
PC1	5.68	63.14	63.14	2.38
PC2	1.21	13.39	76.53	1.10
Acoustic parameters	Coordinates		Contribution	
	PC1	PC2	PC1	PC2
Duration	0.201	-0.118	0.714	1.146
Peak frequency	-0.770	0.419	10.438	14.598
Peak amplitude	0.709	0.600	8.844	29.871
Minimum frequency	-0.393	0.740	2.719	45.487
Maximum frequency	-0.948	-0.109	15.807	0.979
Bandwidth	-0.947	-0.116	15.771	1.124
Quartile 25	-0.927	0.218	15.107	3.950
Quartile 50	-0.951	-0.038	15.909	0.117
Quartile 75	-0.914	-0.181	14.691	2.727

B. PCA analysis on complex sounds pulse rate parameters (Fig. 1i).

	Variance	% of variance	Cumulative % of variance	SD
PC1	2.344	46.887	46.887	1.5311
PC2	1.524	30.472	77.359	1.2343
Acoustic parameters	Coordinates		Contribution	
	PC1	PC2	PC1	PC2
Pulse number	0.84	-0.41	30.43	10.84
Interpulse duration mean	-0.47	-0.62	9.26	25.60
Pulse duration mean	-0.78	-0.11	25.77	0.85
Sound duration	0.55	-0.80	12.77	41.97
Pulse rate	0.71	0.56	21.77	20.74

C. PCA analysis on lab- versus wild- recorded sounds (Fig. 2c).

	**Variance**	**% of variance**	**Cumulative % of variance**	**SD**
PC1	5.25	58.30	58.30	2.30
PC2	1.40	15.50	73.80	1.18
**Acoustic parameters**	**Coordinates**		**Contribution**	
	**PC1**	**PC2**	**PC1**	**PC2**
Duration	0.31	-0.70	1.85	34.93
Peak frequency	-0.78	0.25	11.51	4.34
Peak amplitude	0.71	0.12	9.71	0.97
Minimum frequency	-0.19	0.75	0.66	40.12
Maximum frequency	-0.93	-0.06	16.48	0.25
Bandwidth	-0.93	-0.08	16.37	0.43
Quartile 25	-0.92	0.18	16.06	2.21
Quartile 50	-0.92	-0.16	16.00	1.89
Quartile 75	-0.77	-0.45	11.35	14.88

D. pFDA analysis (Fig. 1h).

Number of permutations	1000	
	Mean (%)	p value
Total of correct classification	76	0,001
Correct classification for random permutation	33	0,001

Supplementary Table 2: Correlation matrix calculated on the resident-intruder assay in surface fish and cavefish.

Data show Pearson's correlation coefficients calculated at one second time bin between sounds and attacks. High correlation coefficient scores are depicted by darker shades of brown on the heat map shown in Figure 4.

**Surface fish**	Single clocs	Serial clocs	Single clicks	Serial clicks	Sharp clicks	Rumblings	Attacks
Single clocs	1.0000	0.0371	-0.0190	-0.0148	-0.0276	0.0028	0.0224
Serial clocs	0.0371	1.0000	-0.0069	0.0030	-0.0006	-0.0140	0.0291
Single clicks	-0.0190	-0.0069	1.0000	0.0972	-0.0124	-0.0101	-0.0206
Serial clicks	-0.0148	0.0030	0.0972	1.0000	0.0594	0.0556	0.0360
Sharp clicks	-0.0276	-0.0006	-0.0124	0.0594	1.0000	0.0537	0.4558
Rumblings	0.0028	-0.0140	-0.0101	0.0556	0.0537	1.0000	0.1424
Attacks	0.0224	0.0291	-0.0206	0.0360	0.4558	0.1424	1.0000
**Cavefish**	Single clocs	Serial clocs	Single clicks	Serial clicks	Sharp clicks	Rumblings	Attacks
Single clocs	1.0000	0.0190	-0.0088	-0.0070	-0.0150	-0.0071	0.0258
Serial clocs	0.0190	1.0000	-0.0064	-0.0151	-0.0019	-0.0012	-0.0097
Single clicks	-0.0088	-0.0064	1.0000	0.0724	-0.0040	-0.0241	-0.0096
Serial clicks	-0.0070	-0.0151	0.0724	1.0000	-0.0094	-0.0138	-0.0078
Sharp clicks	-0.0150	-0.0019	-0.0040	-0.0094	1.0000	-0.0105	0.0473
Rumblings	-0.0071	-0.0012	-0.0241	-0.0138	-0.0105	1.0000	-0.0063
Attacks	0.0258	-0.0097	-0.0096	-0.0078	0.0473	-0.0063	1.0000

Supplementary Table 3: Correlation matrix calculated for the mirror assay in surface fish.

The table shows Pearson's correlation coefficients calculated at one second time bin between sounds, positions and attacks. High correlation coefficient scores are depicted by darker shades of brown on the corresponding heat map shown on Figure 4.

Pearson's correlation coefficients	Single clocs	Serial clocs	Single clicks	Serial clicks	Sharp clicks	Rumblings	Mirror close	Mirror far	Attacks
Single clocs	1.0000	0.0328	-0.0255	-0.0124	-0.0247	-0.0459	0.5553	0.2266	0.0022
Serial clocs	0.0328	1.0000	-0.0101	-0.0116	-0.0196	0.0058	0.4841	0.0888	0.0222
Single clicks	-0.0255	-0.0101	1.0000	-0.0029	-0.0096	-0.0107	0.0846	0.1474	-0.0225
Serial clicks	-0.0124	-0.0116	-0.0029	1.0000	-0.0085	-0.0128	0.0113	0.0892	-0.0114
Sharp clicks	-0.0247	-0.0196	-0.0096	-0.0085	1.0000	0.0961	0.0819	-0.0178	0.6409
Rumblings	-0.0459	0.0058	-0.0107	-0.0128	0.0961	1.0000	0.2971	0.0255	0.4639
Mirror close	0.5553	0.4841	0.0846	0.0113	0.0819	0.2971	1.0000	-0.0743	-0.0126
Mirror far	0.2266	0.0888	0.1474	0.0892	-0.0178	0.0255	-0.0743	1.0000	-0.0546
Attacks	0.0022	0.0222	-0.0225	-0.0114	0.6409	0.4639	-0.0126	-0.0546	1.0000

Supplementary Reference

1. **Schemmel, C.,** ***Z.*** Studies on the genetics of feeding behaviour in the cave fish Astyanax mexicanus f. Anoptichthys. An example of apparent monofactorial inheritance by polygenes. ***Tierpsychol.*** **53, 9-22 (1980).**
