# Supplementary material for: Evolution of acoustic communication in blind cavefish
Source: Nat Commun. 2019 Sep 17;10:4231. doi: 10.1038/s41467-019-12078-9 (PMC6748933; doi:10.1038/s41467-019-12078-9)
Supplement: Supplementary file 3 — Reporting Summary [file 41467_2019_12078_MOESM3_ESM.pdf]

nature research

Corresponding author(s): Sylvie RETAUX

Last updated by author(s): 07/18/2019

Reporting Summary

Nature Research wishes to improve the reproducibility of the work that we publish. This form provides structure for consistency and transparency in reporting. For further information on Nature Research policies, see Authors & Referees and the Editorial Policy Checklist.

Statistics

For all statistical analyses, confirm that the following items are present in the figure legend, table legend, main text, or Methods section.

n/a	Confirmed	
☐	☒	The exact sample size (n) for each experimental group/condition, given as a discrete number and unit of measurement
☐	☒	A statement on whether measurements were taken from distinct samples or whether the same sample was measured repeatedly
☐	☒	The statistical test(s) used AND whether they are one- or two-sided<br>*Only common tests should be described solely by name; describe more complex techniques in the Methods section.*
☒	☐	A description of all covariates tested
☐	☒	A description of any assumptions or corrections, such as tests of normality and adjustment for multiple comparisons
☐	☒	A full description of the statistical parameters including central tendency (e.g. means) or other basic estimates (e.g. regression coefficient) AND variation (e.g. standard deviation) or associated estimates of uncertainty (e.g. confidence intervals)
☐	☒	For null hypothesis testing, the test statistic (e.g. F, t, r) with confidence intervals, effect sizes, degrees of freedom and P value noted<br>*Give P values as exact values whenever suitable.*
☒	☐	For Bayesian analysis, information on the choice of priors and Markov chain Monte Carlo settings
☐	☒	For hierarchical and complex designs, identification of the appropriate level for tests and full reporting of outcomes
☐	☒	Estimates of effect sizes (e.g. Cohen's d, Pearson's r), indicating how they were calculated

Our web collection on statistics for biologists contains articles on many of the points above.

Software and code

Policy information about availability of computer code

Data collection	NA
Data analysis	NA

For manuscripts utilizing custom algorithms or software that are central to the research but not yet described in published literature, software must be made available to editors/reviewers. We strongly encourage code deposition in a community repository (e.g. GitHub). See the Nature Research guidelines for submitting code & software for further information.

Data

Policy information about availability of data

All manuscripts must include a data availability statement. This statement should provide the following information, where applicable:

- Accession codes, unique identifiers, or web links for publicly available datasets
- A list of figures that have associated raw data
- A description of any restrictions on data availability

There is no restriction on data availability. Supplementary Table 1 provides raw data acoustic parameters for 516 sounds used in the analyses.

Field-specific reporting

Please select the one below that is the best fit for your research. If you are not sure, read the appropriate sections before making your selection.

☒ Life sciences ☐ Behavioural & social sciences ☐ Ecological, evolutionary & environmental sciences

For a reference copy of the document with all sections, see nature.com/documents/nr-reporting-summary-flat.pdf

Life sciences study design

All studies must disclose on these points even when the disclosure is negative.

Sample size	No statistical method was used to predetermine sample size. Animals: adult male and female fish aged between 1 and 8 years old (Pachón cavefish and surface fish), sized between 3.5 and 6.5cm, and born in our facility were used. Sample size reached the minimum number needed for each beavioural test, depending on social conditions to be tested, and to perform reliable statistical analyses.<br>Sound samples: a bank of 516 sounds was originaly constituted to perform a screening of acoustic parameters and further multivariate analyses (see Supplementary table1).
Data exclusions	No data were excluded from analysis.
Replication	Each analysis had either biological or technical replicates to get rid off inter or intra-individual differences.
Randomization	Allocation was random.
Blinding	For sound samples data base: blinding was irrelevant as the extraction of sounds in the lab or in the wild had to be performed by a human prior doing non-supervised PCA or supervised pDFA analyses.<br>For behaviour experiments: blinding was not possible as investigators needed to know well the sounds and discriminate between differents sounds of the repertoire, and to watch videos to score both sounds and behaviours simultaneously.

Reporting for specific materials, systems and methods

We require information from authors about some types of materials, experimental systems and methods used in many studies. Here, indicate whether each material, system or method listed is relevant to your study. If you are not sure if a list item applies to your research, read the appropriate section before selecting a response.

Materials & experimental systems

n/a	Involved in the study	
☒	☐	Antibodies
☒	☐	Eukaryotic cell lines
☒	☐	Palaeontology
☐	☒	Animals and other organisms
☒	☐	Human research participants
☒	☐	Clinical data

Methods

n/a	Involved in the study	
☒	☐	ChIP-seq
☒	☐	Flow cytometry
☒	☐	MRI-based neuroimaging

Animals and other organisms

Policy information about studies involving animals; ARRIVE guidelines recommended for reporting animal research

Laboratory animals	Please, see Methods, Fish samples. Laboratory stocks of A. mexicanus surface fish (origin: San Salomon spring, Texas, USA) and cavefish (Pachón population) were obtained in 2004 from the Jeffery laboratory at the University of Maryland, College Park, MD. The breeding colonies were since then maintained at 23° (cavefish) or 26°C (surface fish) on a 12:12 hours light:dark cycle.
Wild animals	Please, see Methods, Fish samples. The animal facility of the Institute received authorization 91272105 from the Veterinary Services of Essonne in 2015. Field recordings were obtained during two field expeditions in the states of San Luis Potosi and Tamaulipas, Mexico, in March 2016 and March 2017, under the auspices of the field permit 02438/16, delivered by the Mexican Secretaria de Medio Ambiente y Recursos Naturales.
Field-collected samples	No animal were collected form field experiments. Experiments were performed on site and all animals were left in their natural niches.
Ethics oversight	Animals were treated according to the French and European regulations for handling of animals in research. SR’s authorization for use of Astyanax mexicanus in research is 91-116 and the Paris Centre-Sud Ethic Committee protocol authorization number related to this work is 2012-0054.

Note that full information on the approval of the study protocol must also be provided in the manuscript.
