# Supplementary material for: Evolution of acoustic communication in blind cavefish
Source: Nat Commun. 2019 Sep 17;10:4231. doi: 10.1038/s41467-019-12078-9 (PMC6748933; doi:10.1038/s41467-019-12078-9)
Supplement: Supplementary file 4 — Description of Additional Supplementary Files [file 41467_2019_12078_MOESM4_ESM.pdf]

Description of Additional Supplementary Files

File Name: Supplementary Data 1
Description: Acoustic parameters of 605 simple sounds of Astyanax mexicanus recorded in the lab (a) and in the wild (b).

a, Acoustic parameters (columns 3 to 11) of 516 sounds of SF (n=10 individuals) and Pachón CF (n=10 individuals). Sound number (#) is in column 1. Individual sound code (column 2) indicates morphotype and sound category with a capital letter A, B (grey) = Single Clocs for SF and CF respectively; C, D (green) = Single Clicks for SF and CF respectively and E, F (yellow) = Sharp Clicks for SF and CF respectively. The first number following a capital letter identifies individuals (from 1 to 10) and the second number (from 1 to 14) specifies sound replicates; e.g. sound number #516 is F106 = Sharp click from CF number 10, replicate number 6.

b, Acoustic parameters (column 3 to 11) of 89 sounds of Pachón CF (n=12 individuals in a net) recorded in the Pachón cave. Sound number (#) is in column 1. Individual sound code (column 2) indicates sound categories with a capital letter X (light grey) = Wild Single Clocs and Y (light green) = Wild Single Clicks. Min = minimum, Max = maximum, s = second, Hz = hertz, dB = decibel.

File Name: Supplementary Data 2
Description: Acoustic parameters (column 3 to 7) of 186 complex sounds recorded in the lab (n=12 SF, n=9 Pachón CF), used for temporal description and pulse rate analysis. Number of sound (#) is in column 1. Individual sound code (column 2) indicates morph and sound categories with a capital letter A, B (light grey) = Serial clocs for SF and CF respectively, C, D (light green) = Serial clicks for SF and CF respectively. Min = minimum, Max = maximum, s = second, Hz = hertz, dB = decibel

File Name: Supplementary Data 3
Description: summary statistics for all graphs presented in the different figures.

File Name: Supplementary Audio 1
Description: single cloc

File Name: Supplementary Audio 2
Description: single click

File Name: Supplementary Audio 3
Description: sharp click

File Name: Supplementary Audio 4
Description: serial cloc

File Name: Supplementary Audio 5
Description: serial click

File Name: Supplementary Audio 6
Description: rumbling

File Name: Supplementary Audio 7
Description: serial click-like from the Molino cave

File Name: Supplementary Audio 8
Description: : serial click-like from the Pachón cave

File Name: Supplementary Audio 9
Description: : serial click-like from wild surface fish

File Name: Supplementary Audio 10
Description: serial click-like from Los Sabinos cave

File Name: Supplementary Audio 11
Description: serial click-like from the Tinaja cave

File Name: Supplementary Audio 12
Description: serial click-like from the Subterráneo cave

File Name: Supplementary Audio 13
Description: serial click-like from the Chica cave

File Name: Supplementary Audio 14
Description: single cloc from the Pachón cave

File Name: Supplementary Audio 15
Description: single click from the Pachón cave

File Name: Supplementary Audio 16
Description: sharp click from the Pachón cave

File Name: Supplementary Audio 17
Description: serial cloc from the Pachón cave

File Name: Supplementary Audio 18
Description: serial click from the Pachón cave

File Name: Supplementary Audio 19
Description: rumbling from the Pachón cave

File Name: Supplementary Movie 1
Description: **concomitant attack and sharp click emission, between two surface fish.**
Video showing the concomitance of attacks/aggressive behavior and the emission of sharp clicks during a SF resident-intruder assay.

File Name: Supplementary Movie 2
Description: **effect of white noise on surface fish.**
Video showing SF behavioral response to white noise. Note that before the stimulation, the dominant fish stands alone in zone O while the subordinates swim in zones S and M. The green oval indicates the zone where all fish gather upon stimulus, while increasing their swimming speed.

File Name: Supplementary Movie 3

Description: **effect of sharp clicks on surface fish.**

Video showing SF behavioral response to Sharp Click playback. Note that before the stimulation, the dominant fish stands alone in zone O while the subordinates swim in zones S and M. The green oval indicates the zone where all fish gather upon stimulus, without increase of swimming speed.

File Name: Supplementary Movie 4

Description: **effect of white noise play-back on cavefish.**

Video showing CF behavioral response to white noise. The stimulus did not elicit a change of place in the aquarium, only an increase in swimming speed.

File Name: Supplementary Movie 5

Description: **effect of sharp click play-back on cavefish.**

Video showing CF behavioral response to Sharp Click playback. Note that before the stimulation, the 6 fish swim in zones O and M. The green oval indicates the zone close to the speaker where they reach upon stimulus, without increasing swimming speed. Note that after the stimulus, 2 fish adopt a foraging posture on the bottom of the tank.
