# Supplementary material for: Evolution of acoustic communication in blind cavefish
Source: Nat Commun. 2019 Sep 17;10:4231. doi: 10.1038/s41467-019-12078-9 (PMC6748933; doi:10.1038/s41467-019-12078-9)
Supplement: Supplementary file 24 — Supplementary Data 1 [file 41467_2019_12078_MOESM24_ESM.doc]

**Supplementary data 1**: Acoustic parameters of 605 simple sounds of *Astyanax mexicanus* recorded in the lab (**a**) and in the wild (**b**).

**a,** Acoustic parameters (columns 3 to 11) of 516 sounds of SF (n=10 individuals) and Pachón CF (n=10 individuals). Sound number (#) is in column 1. Individual sound code (column 2) indicates morphotype and sound category with a capital letter A, B (grey) = Single Clocs for SF and CF respectively; C, D (green) = Single Clicks for SF and CF respectively and E, F (yellow) = Sharp Clicks for SF and CF respectively. The first number following a capital letter identifies individuals (from 1 to 10) and the second number (from 1 to 14) specifies sound replicates; *e.g*. sound number #516 is F106 = Sharp click from CF number 10, replicate number 6.

**b**, Acoustic parameters (column 3 to 11) of 89 sounds of Pachón CF (n=12 individuals in a net) recorded in the Pachón cave. Sound number (#) is in column 1. Individual sound code (column 2) indicates sound categories with a capital letter X (light grey) = Wild Single Clocs and Y (light green) = Wild Single Clicks.

**Min** = minimum, **Max** = maximum, **s** = second, **Hz** = hertz, **dB** = decibel.

1. Lab recorded sounds

| **Sound number (#)** | **Individual**  **sound**  **code** | **Acoustic parameters** | | | | | | | | |
| --- | --- | --- | --- | --- | --- | --- | --- | --- | --- | --- |
|  |  | **Duration**  **(s)** | **Peak frequency (Hz)** | **Peak amplitude**  **(dB)** | **Min frequency (Hz)** | **Max frequency (Hz)** | **Bandwidth** | **Quartile 25** | **Quartile 50** | **Quartile 75** |
| #1 | A11 | 0.01 | 177 | -38.81 | 129 | 861 | 732 | 301 | 559 | 2842 |
| #2 | A12 | 0.01 | 232 | -33.81 | 129 | 602 | 473 | 301 | 473 | 3445 |
| #3 | A13 | 0.02 | 273 | -37.28 | 129 | 1421 | 1292 | 344 | 602 | 1981 |
| #4 | A14 | 0.02 | 174 | -33.32 | 129 | 344 | 215 | 215 | 473 | 5555 |
| #5 | A15 | 0.01 | 189 | -29.5 | 129 | 473 | 344 | 215 | 301 | 1507 |
| #6 | A16 | 0.01 | 188 | -21.5 | 129 | 387 | 258 | 215 | 258 | 344 |
| #7 | A21 | 0.01 | 226 | -43.15 | 129 | 732 | 602 | 301 | 1550 | 6589 |
| #8 | A22 | 0.02 | 297 | -49.23 | 129 | 5211 | 5081 | 516 | 3488 | 9000 |
| #9 | A23 | 0.02 | 252 | -47.87 | 129 | 4694 | 4565 | 473 | 3445 | 8957 |
| #10 | A24 | 0.01 | 237 | -41.47 | 129 | 1636 | 1507 | 344 | 602 | 4263 |
| #11 | A25 | 0.01 | 221 | -49.17 | 172 | 6029 | 5857 | 559 | 3789 | 8828 |
| #12 | A26 | 0.01 | 215 | -39.58 | 129 | 1248 | 1119 | 301 | 818 | 4823 |
| #13 | A27 | 0.01 | 214 | -41.2 | 129 | 861 | 732 | 387 | 602 | 3186 |
| #14 | A28 | 0.01 | 220 | -31.81 | 129 | 861 | 732 | 258 | 430 | 3143 |
| #15 | A29 | 0.01 | 227 | -27.39 | 129 | 387 | 258 | 258 | 301 | 1765 |
| #16 | A31 | 0.02 | 272 | -30.5 | 129 | 861 | 732 | 301 | 387 | 1248 |
| #17 | A32 | 0.01 | 187 | -33.53 | 129 | 430 | 301 | 215 | 344 | 3617 |
| #18 | A33 | 0.01 | 239 | -34.97 | 129 | 904 | 775 | 301 | 387 | 1335 |
| #19 | A34 | 0.02 | 216 | -39.72 | 172 | 861 | 689 | 301 | 1378 | 6632 |
| #20 | A35 | 0.01 | 178 | -35.5 | 129 | 473 | 344 | 215 | 430 | 4694 |
| #21 | A41 | 0.01 | 216 | -37.82 | 129 | 430 | 301 | 258 | 387 | 5900 |
| #22 | A51 | 0.02 | 222 | -39.67 | 129 | 473 | 344 | 301 | 1593 | 6373 |
| #23 | A52 | 0.02 | 190 | -31.76 | 129 | 430 | 301 | 215 | 301 | 2540 |
| #24 | A53 | 0.03 | 194 | -25.91 | 129 | 473 | 344 | 215 | 258 | 430 |
| #25 | A54 | 0.02 | 194 | -42.21 | 129 | 602 | 473 | 344 | 732 | 6115 |
| #26 | A55 | 0.02 | 191 | -40.07 | 129 | 516 | 387 | 258 | 473 | 5254 |
| #27 | A56 | 0.01 | 290 | -38.73 | 129 | 516 | 387 | 344 | 602 | 6158 |
| #28 | A57 | 0.01 | 260 | -25.25 | 129 | 473 | 344 | 258 | 344 | 516 |
| #29 | A58 | 0.01 | 222 | -37.94 | 129 | 559 | 430 | 301 | 473 | 4737 |
| #30 | A59 | 0.02 | 174 | -33.54 | 129 | 344 | 215 | 215 | 301 | 5211 |
| **Sound number (#)** | **Individual**  **sound**  **code** | **Acoustic parameters** | | | | | | | | |
|  |  | **Duration**  **(s)** | **Peak frequency (Hz)** | **Peak amplitude**  **(dB)** | **Min frequency (Hz)** | **Max frequency (Hz)** | **Bandwidth** | **Quartile 25** | **Quartile 50** | **Quartile 75** |
|  |  |  |  |  |  |  |  |  |  |  |
| #31 | A510 | 0.01 | 199 | -25.33 | 129 | 473 | 344 | 215 | 301 | 387 |
| #32 | A61 | 0.03 | 203 | -38.6 | 129 | 387 | 258 | 258 | 990 | 5555 |
| #33 | A62 | 0.01 | 196 | -49.12 | 129 | 6287 | 6158 | 516 | 2411 | 7192 |
| #34 | A63 | 0.03 | 233 | -38.21 | 129 | 387 | 258 | 258 | 1292 | 6072 |
| #35 | A64 | 0.02 | 252 | -37.39 | 129 | 430 | 301 | 301 | 387 | 5469 |
| #36 | A65 | 0.01 | 196 | -49.45 | 129 | 7106 | 6976 | 689 | 3402 | 8957 |
| #37 | A66 | 0.01 | 227 | -40.02 | 129 | 1550 | 1421 | 301 | 689 | 4091 |
| #38 | A71 | 0.02 | 209 | -44.68 | 129 | 1378 | 1248 | 301 | 2756 | 7149 |
| #39 | A72 | 0.01 | 250 | -40.48 | 129 | 473 | 344 | 301 | 1765 | 6158 |
| #40 | A73 | 0.01 | 228 | -44.75 | 129 | 516 | 387 | 344 | 1808 | 6632 |
| #41 | A74 | 0.01 | 245 | -52.8 | 129 | 16193 | 16063 | 1550 | 4478 | 9603 |
| #42 | A75 | 0.03 | 179 | -51.65 | 129 | 7321 | 7192 | 1464 | 4220 | 9345 |
| #43 | A76 | 0.01 | 209 | -42.26 | 129 | 689 | 559 | 301 | 1679 | 6416 |
| #44 | A77 | 0.02 | 175 | -42.48 | 129 | 1593 | 1464 | 258 | 1679 | 6632 |
| #45 | A78 | 0.01 | 263 | -50.64 | 129 | 9259 | 9130 | 602 | 3273 | 8828 |
| #46 | A81 | 0.01 | 333 | -51.65 | 129 | 7235 | 7106 | 646 | 3100 | 7321 |
| #47 | A82 | 0.01 | 195 | -54.93 | 129 | 11369 | 11240 | 1464 | 3962 | 9345 |
| #48 | A83 | 0.01 | 289 | -41.76 | 129 | 1679 | 1550 | 301 | 430 | 4565 |
| #49 | A84 | 0.01 | 168 | -43.57 | 129 | 732 | 602 | 301 | 1119 | 6416 |
| #50 | A85 | 0.01 | 199 | -52.25 | 129 | 10292 | 10163 | 904 | 3832 | 8914 |
| #51 | A86 | 0.01 | 226 | -38.28 | 129 | 516 | 387 | 301 | 430 | 4823 |
| #52 | A87 | 0.01 | 219 | -45.81 | 129 | 1722 | 1593 | 344 | 1851 | 6675 |
| #53 | A88 | 0.01 | 292 | -40.28 | 129 | 516 | 387 | 301 | 430 | 4392 |
| #54 | A89 | 0.01 | 219 | -39.01 | 129 | 646 | 516 | 301 | 473 | 5168 |
| #55 | A810 | 0.01 | 199 | -42.38 | 129 | 602 | 473 | 301 | 1205 | 6373 |
| #56 | A811 | 0.01 | 221 | -43.91 | 129 | 1335 | 1205 | 344 | 2153 | 6503 |
| #57 | A812 | 0.01 | 183 | -42.48 | 129 | 602 | 473 | 344 | 1636 | 5986 |
| #58 | A813 | 0.01 | 224 | -38.79 | 129 | 646 | 516 | 301 | 430 | 4091 |
| #59 | A814 | 0.01 | 222 | -38.94 | 129 | 516 | 387 | 301 | 559 | 5038 |
| #60 | A91 | 0.01 | 200 | -45.85 | 129 | 4823 | 4694 | 344 | 2454 | 6890 |
| #61 | A92 | 0.01 | 263 | -36.32 | 129 | 861 | 732 | 301 | 430 | 1765 |
| #62 | A93 | 0.01 | 161 | -52.97 | 86 | 10379 | 10292 | 1378 | 4478 | 9646 |
| #63 | A94 | 0.01 | 184 | -50.7 | 129 | 6675 | 6546 | 646 | 3789 | 8957 |
| #64 | A95 | 0.01 | 192 | -50.32 | 129 | 5814 | 5684 | 602 | 3359 | 8311 |
| #65 | A96 | 0.01 | 194 | -45.6 | 129 | 4005 | 3876 | 387 | 3014 | 7278 |
| #66 | A97 | 0.01 | 218 | -43.1 | 129 | 646 | 516 | 344 | 1722 | 6201 |
| #67 | A98 | 0.01 | 392 | -42.18 | 129 | 775 | 646 | 430 | 1292 | 6072 |
| #68 | A99 | 0.01 | 226 | -50.77 | 129 | 5900 | 5770 | 1119 | 4651 | 9560 |
| #69 | A910 | 0.01 | 178 | -45.18 | 129 | 430 | 301 | 387 | 3143 | 7407 |
| #70 | A911 | 0.01 | 222 | -38.03 | 129 | 516 | 387 | 258 | 473 | 5383 |
| #71 | A101 | 0.01 | 250 | -29.68 | 129 | 646 | 516 | 258 | 344 | 818 |
| **Sound number (#)** | **Individual**  **sound**  **code** | **Acoustic parameters** | | | | | | | | |
|  |  | **Duration**  **(s)** | **Peak frequency (Hz)** | **Peak amplitude**  **(dB)** | **Min frequency (Hz)** | **Max frequency (Hz)** | **Bandwidth** | **Quartile 25** | **Quartile 50** | **Quartile 75** |
| #72 | A102 | 0.01 | 219 | -38.25 | 129 | 646 | 516 | 258 | 516 | 5168 |
| #73 | A103 | 0.01 | 234 | -51.73 | 172 | 6675 | 6503 | 1033 | 4349 | 9388 |
| #74 | A104 | 0.01 | 264 | -45.46 | 129 | 5512 | 5383 | 344 | 1679 | 6503 |
| #75 | A105 | 0.01 | 191 | -45.81 | 129 | 4306 | 4177 | 387 | 3359 | 8828 |
| #76 | A106 | 0.01 | 250 | -54.32 | 129 | 9948 | 9819 | 1292 | 3617 | 9130 |
| #77 | A107 | 0.02 | 199 | -44.37 | 129 | 559 | 430 | 344 | 2713 | 7881 |
| #78 | A108 | 0.01 | 233 | -43.34 | 129 | 2239 | 2110 | 344 | 1679 | 6416 |
| #79 | A109 | 0.02 | 232 | -45.78 | 129 | 1205 | 1076 | 301 | 2799 | 6890 |
| #80 | A1010 | 0.02 | 259 | -43.82 | 129 | 2024 | 1894 | 344 | 2282 | 7062 |
| #81 | A1011 | 0.01 | 229 | -36.01 | 129 | 602 | 473 | 258 | 387 | 3359 |
| #82 | A1012 | 0.01 | 228 | -42.21 | 129 | 1722 | 1593 | 344 | 861 | 5598 |
| #83 | B11 | 0.02 | 210 | -39.72 | 120 | 1550 | 1420 | 300 | 1240 | 6200 |
| #84 | B12 | 0.02 | 170 | -33.38 | 120 | 380 | 250 | 210 | 300 | 3480 |
| #85 | B13 | 0.03 | 189 | -35.33 | 129 | 904 | 775 | 301 | 602 | 3359 |
| #86 | B14 | 0.01 | 260 | -47.33 | 129 | 3014 | 2885 | 473 | 2799 | 8871 |
| #87 | B15 | 0.01 | 181 | -38.44 | 129 | 516 | 387 | 258 | 473 | 5598 |
| #88 | B16 | 0.01 | 208 | -38.82 | 129 | 559 | 430 | 258 | 473 | 5081 |
| #89 | B17 | 0.01 | 194 | -29.96 | 129 | 818 | 689 | 258 | 473 | 947 |
| #90 | B18 | 0.01 | 181 | -30.44 | 129 | 387 | 258 | 215 | 301 | 3402 |
| #91 | B19 | 0.01 | 208 | -28.45 | 129 | 473 | 344 | 215 | 301 | 861 |
| #92 | B21 | 0.01 | 210 | -36.83 | 120 | 430 | 300 | 250 | 380 | 4780 |
| #93 | B22 | 0.01 | 210 | -37.52 | 120 | 470 | 340 | 300 | 770 | 5510 |
| #94 | B23 | 0.02 | 250 | -43.07 | 120 | 990 | 860 | 340 | 940 | 6110 |
| #95 | B24 | 0.02 | 250 | -33.28 | 120 | 430 | 300 | 250 | 340 | 4900 |
| #96 | B25 | 0.02 | 170 | -30.35 | 120 | 550 | 430 | 210 | 300 | 900 |
| #97 | B26 | 0.01 | 170 | -40.52 | 120 | 550 | 430 | 250 | 1240 | 6240 |
| #98 | B27 | 0.01 | 250 | -34.46 | 120 | 430 | 300 | 300 | 380 | 4600 |
| #99 | B28 | 0.01 | 210 | -25.31 | 120 | 510 | 380 | 250 | 340 | 470 |
| #100 | B29 | 0.01 | 210 | -26.8 | 120 | 510 | 380 | 250 | 300 | 510 |
| #101 | B210 | 0.01 | 202 | -42.43 | 129 | 473 | 344 | 301 | 2282 | 7493 |
| #102 | B211 | 0.01 | 180 | -36.25 | 129 | 559 | 430 | 215 | 430 | 4220 |
| #103 | B31 | 0.02 | 171 | -43.02 | 129 | 387 | 258 | 301 | 3230 | 8828 |
| #104 | B32 | 0.01 | 277 | -42.04 | 129 | 775 | 646 | 344 | 904 | 5943 |
| #105 | B33 | 0.03 | 209 | -43.51 | 129 | 904 | 775 | 301 | 1292 | 6460 |
| #106 | B34 | 0.01 | 191 | -42.02 | 129 | 559 | 430 | 301 | 2799 | 7235 |
| #107 | B35 | 0.02 | 220 | -43.37 | 129 | 4005 | 3876 | 301 | 1894 | 6675 |
| #108 | B36 | 0.01 | 217 | -34.82 | 129 | 1205 | 1076 | 258 | 473 | 5038 |
| #109 | B37 | 0.01 | 254 | -42.99 | 129 | 2110 | 1981 | 344 | 1248 | 5770 |
| #110 | B38 | 0.02 | 264 | -37.89 | 129 | 516 | 387 | 301 | 1119 | 6201 |
| #111 | B39 | 0.02 | 191 | -36.25 | 129 | 775 | 646 | 301 | 430 | 2842 |
| #112 | B310 | 0.02 | 184 | -37.23 | 129 | 473 | 344 | 258 | 430 | 5340 |
| #113 | B311 | 0.02 | 202 | -31.08 | 129 | 387 | 258 | 215 | 301 | 3273 |
| **Sound number (#)** | **Individual**  **sound**  **code** | **Acoustic parameters** | | | | | | | | |
|  |  | **Duration**  **(s)** | **Peak frequency (Hz)** | **Peak amplitude**  **(dB)** | **Min frequency (Hz)** | **Max frequency (Hz)** | **Bandwidth** | **Quartile 25** | **Quartile 50** | **Quartile 75** |
| #114 | B41 | 0.01 | 210 | -31.58 | 120 | 380 | 250 | 250 | 300 | 3180 |
| #115 | B42 | 0.02 | 170 | -44.18 | 120 | 1720 | 1590 | 640 | 3660 | 9130 |
| #116 | B43 | 0.01 | 170 | -46.23 | 120 | 4040 | 3910 | 380 | 1200 | 6330 |
| #117 | B44 | 0.02 | 210 | -42.89 | 120 | 380 | 250 | 300 | 3660 | 9560 |
| #118 | B45 | 0.01 | 248 | -38.03 | 172 | 516 | 344 | 301 | 732 | 5814 |
| #119 | B46 | 0.01 | 198 | -35.65 | 129 | 473 | 344 | 258 | 344 | 4177 |
| #120 | B47 | 0.01 | 220 | -44.71 | 129 | 1292 | 1162 | 301 | 2196 | 6933 |
| #121 | B48 | 0.02 | 192 | -51.58 | 129 | 10249 | 10120 | 1162 | 4349 | 9474 |
| #122 | B49 | 0.01 | 207 | -38.77 | 129 | 646 | 516 | 258 | 344 | 4823 |
| #123 | B410 | 0.01 | 205 | -32.69 | 129 | 516 | 387 | 258 | 301 | 3359 |
| #124 | B411 | 0.01 | 227 | -44.09 | 129 | 1938 | 1808 | 301 | 1851 | 6675 |
| #125 | B51 | 0.01 | 170 | -30.11 | 120 | 380 | 250 | 210 | 300 | 3100 |
| #126 | B52 | 0.02 | 210 | -34.99 | 120 | 510 | 380 | 250 | 340 | 3700 |
| #127 | B53 | 0.01 | 210 | -35.78 | 120 | 430 | 300 | 250 | 340 | 4780 |
| #128 | B54 | 0.03 | 197 | -31.01 | 129 | 473 | 344 | 215 | 301 | 2540 |
| #129 | B55 | 0.01 | 210 | -23.54 | 170 | 430 | 250 | 250 | 300 | 550 |
| #130 | B56 | 0.01 | 170 | -21.2 | 120 | 380 | 250 | 210 | 250 | 380 |
| #131 | B57 | 0.02 | 170 | -34.99 | 120 | 430 | 300 | 250 | 340 | 3140 |
| #132 | B61 | 0.02 | 219 | -31.25 | 129 | 516 | 387 | 258 | 344 | 1894 |
| #133 | B62 | 0.02 | 169 | -31.81 | 129 | 473 | 344 | 215 | 301 | 3445 |
| #134 | B63 | 0.01 | 229 | -36.65 | 129 | 430 | 301 | 258 | 387 | 5426 |
| #135 | B64 | 0.04 | 162 | -40.36 | 129 | 387 | 258 | 215 | 2196 | 7019 |
| #136 | B65 | 0.03 | 168 | -38.94 | 129 | 1248 | 1119 | 258 | 990 | 6158 |
| #137 | B66 | 0.05 | 176 | -43.94 | 86 | 3316 | 3230 | 301 | 3230 | 8785 |
| #138 | B67 | 0.02 | 188 | -38.16 | 129 | 516 | 387 | 258 | 646 | 5814 |
| #139 | B68 | 0.02 | 197 | -31.94 | 129 | 387 | 258 | 215 | 301 | 3402 |
| #140 | B71 | 0.03 | 170 | -32.1 | 120 | 470 | 340 | 210 | 300 | 2920 |
| #141 | B72 | 0.03 | 170 | -29.03 | 120 | 380 | 250 | 210 | 300 | 2020 |
| #142 | B73 | 0.02 | 170 | -33.91 | 120 | 340 | 210 | 210 | 510 | 5210 |
| #143 | B74 | 0.01 | 170 | -23.48 | 120 | 340 | 210 | 210 | 250 | 340 |
| #144 | B75 | 0.02 | 170 | -41.55 | 120 | 470 | 340 | 250 | 1720 | 7060 |
| #145 | B76 | 0.02 | 210 | -24.65 | 120 | 340 | 210 | 210 | 250 | 430 |
| #146 | B81 | 0.02 | 250 | -40.02 | 120 | 640 | 510 | 300 | 600 | 6110 |
| #147 | B82 | 0.02 | 170 | -38.01 | 120 | 380 | 250 | 210 | 1800 | 6710 |
| #148 | B83 | 0.02 | 210 | -36.65 | 120 | 380 | 250 | 250 | 380 | 5030 |
| #149 | B84 | 0.01 | 170 | -26.31 | 120 | 430 | 300 | 210 | 300 | 1460 |
| #150 | B85 | 0.01 | 170 | -31.34 | 120 | 340 | 210 | 210 | 250 | 3310 |
| #151 | B86 | 0.03 | 250 | -34.48 | 120 | 380 | 250 | 300 | 340 | 4900 |
| #152 | B87 | 0.01 | 210 | -29.85 | 120 | 510 | 380 | 250 | 380 | 1590 |
| #153 | B88 | 0.02 | 170 | -32 | 120 | 470 | 340 | 210 | 300 | 2620 |
| #154 | B91 | 0.01 | 213 | -18.56 | 129 | 990 | 861 | 301 | 473 | 818 |
| #155 | B92 | 0.01 | 208 | -31.65 | 129 | 387 | 258 | 215 | 301 | 2842 |
| **Sound number (#)** | **Individual**  **sound**  **code** | **Acoustic parameters** | | | | | | | | |
|  |  | **Duration**  **(s)** | **Peak frequency (Hz)** | **Peak amplitude**  **(dB)** | **Min frequency (Hz)** | **Max frequency (Hz)** | **Bandwidth** | **Quartile 25** | **Quartile 50** | **Quartile 75** |
| #156 | B93 | 0.01 | 190 | -37.04 | 129 | 516 | 387 | 258 | 430 | 4522 |
| #157 | B94 | 0.02 | 189 | -38.19 | 129 | 387 | 258 | 258 | 602 | 5340 |
| #158 | B95 | 0.01 | 196 | -35.76 | 129 | 1205 | 1076 | 258 | 430 | 2282 |
| #159 | B96 | 0.01 | 203 | -35.3 | 129 | 387 | 258 | 258 | 301 | 4005 |
| #160 | B97 | 0.02 | 170 | -38.01 | 129 | 602 | 473 | 215 | 1464 | 6589 |
| #161 | B98 | 0.02 | 200 | -42.73 | 129 | 473 | 344 | 301 | 2239 | 7062 |
| #162 | B99 | 0.01 | 224 | -32.42 | 129 | 473 | 344 | 258 | 344 | 3143 |
| #163 | B910 | 0.01 | 204 | -36.47 | 129 | 387 | 258 | 258 | 301 | 4952 |
| #164 | B911 | 0.01 | 268 | -41.6 | 129 | 559 | 430 | 344 | 559 | 5512 |
| #165 | B101 | 0.02 | 210 | -36.35 | 120 | 600 | 470 | 250 | 380 | 3480 |
| #166 | B102 | 0.01 | 210 | -27.84 | 120 | 510 | 380 | 210 | 300 | 810 |
| #167 | B103 | 0.03 | 210 | -34.56 | 120 | 510 | 380 | 250 | 340 | 1160 |
| #168 | B104 | 0.03 | 170 | -33.21 | 120 | 300 | 170 | 210 | 640 | 5590 |
| #169 | B105 | 0.01 | 210 | -39.12 | 120 | 510 | 380 | 250 | 470 | 5900 |
| #170 | B106 | 0.02 | 170 | -37.49 | 120 | 380 | 250 | 250 | 1160 | 5900 |
| #171 | C11 | 0.01 | 6506 | -35.24 | 344 | 21963 | 21619 | 4823 | 6804 | 11412 |
| #172 | C12 | 0.01 | 6588 | -27.84 | 43 | 21963 | 21920 | 4780 | 7321 | 11068 |
| #173 | C13 | 0.01 | 6543 | -29.49 | 43 | 21963 | 21920 | 3273 | 7106 | 9991 |
| #174 | C14 | 0.01 | 6590 | -33.46 | 258 | 21920 | 21662 | 5426 | 7622 | 12015 |
| #175 | C15 | 0.01 | 6625 | -54.52 | 172 | 21963 | 21791 | 3531 | 6718 | 10809 |
| #176 | C16 | 0.01 | 1851 | -40.95 | 473 | 18131 | 17657 | 1593 | 2842 | 9819 |
| #177 | C21 | 0.01 | 799 | -56.04 | 172 | 21920 | 21748 | 2497 | 6503 | 11240 |
| #178 | C22 | 0.01 | 807 | -56.41 | 129 | 21102 | 20973 | 1894 | 5254 | 9819 |
| #179 | C23 | 0.01 | 973 | -56.92 | 129 | 21963 | 21834 | 2842 | 6546 | 10120 |
| #180 | C31 | 0.01 | 2019 | -47.29 | 172 | 18174 | 18001 | 2024 | 3273 | 10379 |
| #181 | C32 | 0.01 | 1980 | -52.64 | 172 | 20542 | 20370 | 2024 | 3789 | 10163 |
| #182 | C33 | 0.01 | 6545 | -52.72 | 129 | 21963 | 21834 | 3273 | 6632 | 10637 |
| #183 | C34 | 0.01 | 6513 | -46.99 | 129 | 21963 | 21834 | 3488 | 6675 | 12187 |
| #184 | C35 | 0.01 | 1151 | -45.28 | 258 | 21877 | 21619 | 2153 | 6158 | 11154 |
| #185 | C36 | 0.01 | 6592 | -47.97 | 301 | 21963 | 21662 | 3230 | 6546 | 10895 |
| #186 | C37 | 0.01 | 1941 | -42.23 | 301 | 21705 | 21404 | 1981 | 4435 | 10637 |
| #187 | C38 | 0.02 | 6548 | -29.51 | 129 | 21877 | 21748 | 4349 | 6976 | 12704 |
| #188 | C39 | 0.04 | 6549 | -49.28 | 344 | 21963 | 21619 | 4392 | 7062 | 12532 |
| #189 | C41 | 0.04 | 367 | -54.82 | 129 | 19422 | 19293 | 1851 | 4478 | 10120 |
| #190 | C42 | 0.01 | 395 | -55.36 | 129 | 21920 | 21791 | 2239 | 4909 | 9991 |
| #191 | C43 | 0.03 | 940 | -53.66 | 172 | 21662 | 21490 | 1507 | 4478 | 9646 |
| #192 | C44 | 0.01 | 1266 | -41.53 | 559 | 14814 | 14255 | 1378 | 2024 | 7838 |
| #193 | C45 | 0.01 | 880 | -54.93 | 129 | 21447 | 21317 | 2799 | 6589 | 11326 |
| #194 | C46 | 0.01 | 1205 | -50.26 | 129 | 21662 | 21533 | 1938 | 3746 | 11283 |
| #195 | C47 | 0.01 | 1816 | -56.53 | 215 | 21963 | 21748 | 1722 | 3186 | 9603 |
| #196 | C48 | 0.01 | 990 | -29.29 | 344 | 21705 | 21360 | 1851 | 6287 | 9819 |
| #197 | C49 | 0.01 | 6585 | -42.76 | 215 | 21963 | 21748 | 4909 | 7106 | 12919 |
| **Sound number (#)** | **Individual**  **sound**  **code** | **Acoustic parameters** | | | | | | | | |
|  |  | **Duration**  **(s)** | **Peak frequency (Hz)** | **Peak amplitude**  **(dB)** | **Min frequency (Hz)** | **Max frequency (Hz)** | **Bandwidth** | **Quartile 25** | **Quartile 50** | **Quartile 75** |
| #198 | C410 | 0.02 | 645 | -51.58 | 129 | 21920 | 21791 | 2239 | 6546 | 10594 |
| #199 | C411 | 0.01 | 909 | -51.37 | 258 | 20973 | 20714 | 1033 | 1851 | 8441 |
| #200 | C412 | 0.01 | 341 | -50.64 | 172 | 21447 | 21274 | 1894 | 3962 | 10206 |
| #201 | C51 | 0.01 | 6344 | -43.21 | 258 | 21963 | 21705 | 5943 | 6933 | 11584 |
| #202 | C52 | 0.01 | 6545 | -38.94 | 387 | 21877 | 21490 | 5168 | 8397 | 11929 |
| #203 | C53 | 0.01 | 185 | -50.39 | 129 | 5168 | 5038 | 1335 | 3488 | 8957 |
| #204 | C54 | 0.01 | 3308 | -55.14 | 258 | 21662 | 21404 | 2239 | 4780 | 11068 |
| #205 | C55 | 0.01 | 1159 | -39.45 | 129 | 2325 | 2196 | 1076 | 1550 | 5641 |
| #206 | C56 | 0.01 | 6537 | -38 | 947 | 21920 | 20973 | 5168 | 7536 | 12446 |
| #207 | C57 | 0.01 | 230 | -53.31 | 129 | 21791 | 21662 | 1550 | 3962 | 9603 |
| #208 | C61 | 0.03 | 6419 | -49.62 | 172 | 21963 | 21791 | 5383 | 6847 | 10680 |
| #209 | C62 | 0.01 | 6502 | -45.96 | 172 | 21963 | 21791 | 5555 | 6933 | 10895 |
| #210 | C63 | 0.01 | 6497 | -48.75 | 215 | 21963 | 21748 | 4651 | 7192 | 11541 |
| #211 | C64 | 0.04 | 2111 | -50.57 | 129 | 21963 | 21834 | 2799 | 6373 | 10895 |
| #212 | C65 | 0.01 | 1985 | -57.46 | 129 | 21963 | 21834 | 2239 | 5598 | 9776 |
| #213 | C66 | 0.01 | 6508 | -50.9 | 172 | 21963 | 21791 | 5081 | 6804 | 10723 |
| #214 | C67 | 0.01 | 6548 | -48.8 | 258 | 21963 | 21705 | 3100 | 6847 | 10335 |
| #215 | C68 | 0.01 | 6554 | -53.14 | 129 | 21963 | 21834 | 5211 | 8785 | 12661 |
| #216 | C69 | 0.03 | 689 | -54.42 | 129 | 21705 | 21576 | 1550 | 4952 | 9776 |
| #217 | C610 | 0.01 | 6463 | -52.4 | 129 | 21920 | 21791 | 3186 | 6718 | 11800 |
| #218 | C611 | 0.01 | 270 | -52.56 | 129 | 19121 | 18992 | 2239 | 4565 | 10206 |
| #219 | C612 | 0.01 | 2150 | -34.31 | 301 | 21963 | 21662 | 4177 | 6761 | 12187 |
| #220 | C71 | 0.01 | 1730 | -52.32 | 129 | 21963 | 21834 | 2713 | 5727 | 11584 |
| #221 | C72 | 0.01 | 1986 | -48.85 | 129 | 21834 | 21705 | 2239 | 3574 | 10034 |
| #222 | C73 | 0.01 | 383 | -51.73 | 129 | 13738 | 13609 | 1378 | 3531 | 9173 |
| #223 | C74 | 0.01 | 1760 | -53.14 | 129 | 21274 | 21145 | 1808 | 3402 | 9388 |
| #224 | C75 | 0.01 | 1800 | -55.69 | 172 | 21274 | 21102 | 1851 | 4435 | 9905 |
| #225 | C76 | 0.01 | 6316 | -55.25 | 129 | 21963 | 21834 | 4608 | 7019 | 11068 |
| #226 | C77 | 0.01 | 6431 | -55.81 | 129 | 21963 | 21834 | 4995 | 6804 | 11068 |
| #227 | C78 | 0.01 | 6677 | -47.55 | 172 | 21963 | 21791 | 4995 | 6976 | 11111 |
| #228 | C79 | 0.01 | 1699 | -55.69 | 129 | 21748 | 21619 | 1722 | 3703 | 9560 |
| #229 | C710 | 0.01 | 3731 | -50.45 | 129 | 21963 | 21834 | 3316 | 6373 | 11240 |
| #230 | C81 | 0.02 | 214 | -56.79 | 129 | 21963 | 21834 | 2067 | 5168 | 9948 |
| #231 | C82 | 0.01 | 1719 | -43.12 | 215 | 14384 | 14168 | 1550 | 2110 | 7192 |
| #232 | C83 | 0.01 | 1547 | -58.35 | 129 | 21963 | 21834 | 2110 | 5512 | 10163 |
| #233 | C84 | 0.01 | 752 | -55.69 | 129 | 21963 | 21834 | 2928 | 6675 | 11671 |
| #234 | C85 | 0.01 | 649 | -57.75 | 129 | 21360 | 21231 | 2368 | 5986 | 10077 |
| #235 | C86 | 0.01 | 470 | -54.93 | 129 | 21877 | 21748 | 1464 | 4694 | 9991 |
| #236 | C87 | 0.01 | 6371 | -36.72 | 947 | 21963 | 21016 | 5124 | 6933 | 12446 |
| #237 | C91 | 0.01 | 185 | -56.92 | 129 | 21404 | 21274 | 2153 | 5254 | 10508 |
| #238 | C92 | 0.03 | 1139 | -56.41 | 129 | 21920 | 21791 | 1679 | 5168 | 10077 |
| #239 | C93 | 0.02 | 781 | -52.17 | 129 | 12532 | 12403 | 1033 | 2670 | 8957 |
| **Sound number (#)** | **Individual**  **sound**  **code** | **Acoustic parameters** | | | | | | | | |
|  |  | **Duration**  **(s)** | **Peak frequency (Hz)** | **Peak amplitude**  **(dB)** | **Min frequency (Hz)** | **Max frequency (Hz)** | **Bandwidth** | **Quartile 25** | **Quartile 50** | **Quartile 75** |
| #240 | C94 | 0.01 | 1029 | -55.69 | 172 | 21963 | 21791 | 1421 | 3359 | 9474 |
| #241 | C95 | 0.01 | 1842 | -55.14 | 215 | 14341 | 14125 | 1808 | 3531 | 10077 |
| #242 | C101 | 0.01 | 1938 | -51.73 | 172 | 14298 | 14125 | 1808 | 2885 | 8957 |
| #243 | C102 | 0.01 | 7059 | -41.44 | 559 | 21963 | 21404 | 5469 | 7278 | 12187 |
| #244 | C103 | 0.04 | 1251 | -54.62 | 129 | 21360 | 21231 | 1593 | 3014 | 9474 |
| #245 | C104 | 0.01 | 729 | -48.5 | 129 | 21963 | 21834 | 2024 | 5297 | 10249 |
| #246 | C105 | 0.01 | 1676 | -52.02 | 129 | 21576 | 21447 | 2497 | 6761 | 10422 |
| #247 | C106 | 0.01 | 822 | -56.28 | 129 | 21963 | 21834 | 2497 | 5555 | 9862 |
| #248 | D11 | 0.01 | 1670 | -45.35 | 120 | 17910 | 17780 | 1590 | 2280 | 9040 |
| #249 | D12 | 0.01 | 6580 | -45.81 | 340 | 21960 | 21610 | 6280 | 8780 | 13390 |
| #250 | D13 | 0.01 | 730 | -48.06 | 120 | 21960 | 21830 | 2710 | 5940 | 10290 |
| #251 | D14 | 0.01 | 6540 | -51.3 | 170 | 21920 | 21740 | 5380 | 7060 | 12050 |
| #252 | D15 | 0.01 | 6580 | -50.96 | 170 | 21870 | 21700 | 4820 | 7100 | 12360 |
| #253 | D16 | 0.01 | 2970 | -51.44 | 120 | 21830 | 21700 | 2020 | 3400 | 9940 |
| #254 | D17 | 0.01 | 6595 | -51.73 | 172 | 21920 | 21748 | 5900 | 8828 | 13092 |
| #255 | D18 | 0.01 | 6545 | -51.03 | 172 | 21963 | 21791 | 4995 | 6804 | 10723 |
| #256 | D21 | 0.01 | 1720 | -52.4 | 120 | 18000 | 17870 | 1630 | 2490 | 9250 |
| #257 | D22 | 0.01 | 600 | -52.25 | 120 | 11110 | 10980 | 1370 | 5160 | 10200 |
| #258 | D23 | 0.01 | 730 | -54.93 | 120 | 21870 | 21740 | 2280 | 5900 | 10160 |
| #259 | D24 | 0.01 | 640 | -54.72 | 170 | 21960 | 21790 | 1890 | 5340 | 9730 |
| #260 | D25 | 0.01 | 900 | -52.48 | 120 | 21960 | 21830 | 1980 | 5680 | 9810 |
| #261 | D31 | 0.01 | 1729 | -52.72 | 172 | 18087 | 17915 | 1593 | 2110 | 9000 |
| #262 | D32 | 0.01 | 1002 | -54.62 | 172 | 14599 | 14427 | 1248 | 2368 | 8225 |
| #263 | D33 | 0.01 | 2798 | -35.52 | 387 | 19595 | 19207 | 2540 | 3445 | 12661 |
| #264 | D34 | 0.02 | 1740 | -28.56 | 602 | 14384 | 13781 | 1765 | 2540 | 9689 |
| #265 | D35 | 0.01 | 337 | -55.81 | 129 | 21963 | 21834 | 2411 | 4909 | 9991 |
| #266 | D36 | 0.01 | 267 | -56.53 | 129 | 21963 | 21834 | 2584 | 5512 | 10335 |
| #267 | D37 | 0.01 | 198 | -57.05 | 129 | 21490 | 21360 | 1765 | 4651 | 9388 |
| #268 | D38 | 0.01 | 182 | -55.58 | 129 | 11843 | 11714 | 1248 | 3746 | 9302 |
| #269 | D39 | 0.01 | 1998 | -46.95 | 215 | 18131 | 17915 | 1765 | 2325 | 9302 |
| #270 | D310 | 0.01 | 1328 | -44.55 | 344 | 14771 | 14427 | 1292 | 1765 | 7062 |
| #271 | D311 | 0.01 | 1509 | -55.14 | 129 | 14599 | 14470 | 1507 | 2540 | 8182 |
| #272 | D312 | 0.01 | 2241 | -48.01 | 172 | 20930 | 20758 | 2584 | 4048 | 12101 |
| #273 | D313 | 0.01 | 1803 | -40.38 | 559 | 17872 | 17312 | 2153 | 4263 | 11068 |
| #274 | D314 | 0.01 | 1862 | -53.75 | 172 | 21920 | 21748 | 2196 | 4608 | 10723 |
| #275 | D41 | 0.01 | 1500 | -47.83 | 210 | 14590 | 14380 | 1330 | 2020 | 8350 |
| #276 | D42 | 0.01 | 730 | -48.35 | 120 | 21960 | 21830 | 2620 | 5850 | 10290 |
| #277 | D43 | 0.01 | 600 | -55.47 | 120 | 21830 | 21700 | 2410 | 6070 | 10590 |
| #278 | D44 | 0.01 | 600 | -58.19 | 120 | 21790 | 21660 | 2150 | 5250 | 9940 |
| #279 | D45 | 0.01 | 943 | -53.94 | 129 | 21920 | 21791 | 3100 | 6373 | 10163 |
| #280 | D46 | 0.01 | 6505 | -59.51 | 258 | 21963 | 21705 | 3014 | 6503 | 10379 |
| #281 | D47 | 0.01 | 319 | -58.66 | 172 | 21920 | 21748 | 2713 | 6287 | 10163 |
| **Sound number (#)** | **Individual**  **sound**  **code** | **Acoustic parameters** | | | | | | | | |
|  |  | **Duration**  **(s)** | **Peak frequency (Hz)** | **Peak amplitude**  **(dB)** | **Min frequency (Hz)** | **Max frequency (Hz)** | **Bandwidth** | **Quartile 25** | **Quartile 50** | **Quartile 75** |
| #282 | D48 | 0.01 | 6422 | -49.67 | 172 | 21963 | 21791 | 5211 | 7062 | 11283 |
| #283 | D49 | 0.01 | 6578 | -54.42 | 129 | 21920 | 21791 | 3445 | 6675 | 11068 |
| #284 | D410 | 0.01 | 6549 | -53.05 | 258 | 21963 | 21705 | 5598 | 7536 | 12919 |
| #285 | D411 | 0.01 | 6384 | -53.22 | 129 | 21963 | 21834 | 4952 | 6933 | 11843 |
| #286 | D51 | 0.01 | 1800 | -47.64 | 120 | 17950 | 17820 | 1370 | 2110 | 8350 |
| #287 | D52 | 0.01 | 510 | -56.16 | 170 | 21870 | 21700 | 2450 | 6630 | 10500 |
| #288 | D53 | 0.01 | 510 | -56.28 | 120 | 21870 | 21740 | 3660 | 6840 | 11020 |
| #289 | D54 | 0.01 | 1550 | -51.1 | 120 | 21830 | 21700 | 2320 | 5120 | 11280 |
| #290 | D55 | 0.01 | 250 | -43.68 | 170 | 5340 | 5160 | 1200 | 4900 | 9900 |
| #291 | D56 | 0.01 | 210 | -48.3 | 120 | 21960 | 21830 | 1500 | 6150 | 9810 |
| #292 | D57 | 0.01 | 210 | -46.99 | 120 | 6540 | 6410 | 430 | 3270 | 8950 |
| #293 | D58 | 0.01 | 210 | -49.96 | 120 | 4560 | 4430 | 940 | 3480 | 8820 |
| #294 | D61 | 0.02 | 1290 | -49.96 | 120 | 18770 | 18640 | 1420 | 2750 | 9080 |
| #295 | D62 | 0.01 | 170 | -47.78 | 120 | 3960 | 3830 | 1110 | 3010 | 8520 |
| #296 | D63 | 0.01 | 1160 | -52.48 | 170 | 10290 | 10120 | 1240 | 2540 | 7270 |
| #297 | D64 | 0.01 | 990 | -55.36 | 120 | 14770 | 14640 | 1290 | 2750 | 8220 |
| #298 | D65 | 0.01 | 1240 | -38.36 | 340 | 2110 | 1760 | 1290 | 1590 | 7360 |
| #299 | D66 | 0.01 | 1850 | -46.58 | 120 | 18340 | 18210 | 1070 | 1980 | 6580 |
| #300 | D67 | 0.01 | 1890 | -45.6 | 340 | 18640 | 18300 | 2190 | 4090 | 11320 |
| #301 | D68 | 0.01 | 2790 | -29.13 | 1030 | 19290 | 18260 | 2540 | 3400 | 12740 |
| #302 | D69 | 0.01 | 1500 | -50.02 | 120 | 17610 | 17480 | 1500 | 1980 | 8780 |
| #303 | D71 | 0.02 | 1760 | -53.05 | 120 | 17950 | 17820 | 1420 | 1980 | 7830 |
| #304 | D72 | 0.01 | 6670 | -52.4 | 120 | 21700 | 21570 | 4690 | 7010 | 11240 |
| #305 | D73 | 0.02 | 210 | -44.46 | 120 | 2020 | 1890 | 1110 | 1890 | 7100 |
| #306 | D74 | 0.01 | 340 | -56.28 | 120 | 21740 | 21610 | 2150 | 4900 | 9940 |
| #307 | D75 | 0.01 | 380 | -55.25 | 120 | 21660 | 21530 | 2540 | 5810 | 10590 |
| #308 | D76 | 0.01 | 1110 | -46.95 | 120 | 13600 | 13470 | 1160 | 2110 | 8870 |
| #309 | D77 | 0.01 | 510 | -55.58 | 120 | 21960 | 21830 | 2580 | 6020 | 9860 |
| #310 | D81 | 0.01 | 380 | -54.72 | 120 | 21140 | 21010 | 1460 | 2840 | 9380 |
| #311 | D82 | 0.01 | 6458 | -44.98 | 301 | 21963 | 21662 | 3746 | 6847 | 12144 |
| #312 | D83 | 0.01 | 6545 | -28.79 | 602 | 21963 | 21360 | 4048 | 6847 | 12058 |
| #313 | D84 | 0.01 | 1078 | -48.5 | 258 | 21834 | 21576 | 1679 | 5469 | 9733 |
| #314 | D85 | 0.01 | 6895 | -56.04 | 215 | 21963 | 21748 | 3100 | 6589 | 10292 |
| #315 | D86 | 0.01 | 1800 | -52.48 | 120 | 21310 | 21180 | 1890 | 5720 | 9990 |
| #316 | D87 | 0.01 | 1590 | -54.72 | 210 | 18040 | 17820 | 1670 | 2970 | 9380 |
| #317 | D88 | 0.01 | 1590 | -52.4 | 120 | 18640 | 18510 | 1670 | 2920 | 9640 |
| #318 | D89 | 0.01 | 2060 | -56.66 | 120 | 19460 | 19330 | 1980 | 2970 | 9430 |
| #319 | D810 | 0.01 | 609 | -52.64 | 129 | 21533 | 21404 | 2971 | 5469 | 10938 |
| #320 | D811 | 0.01 | 913 | -54.32 | 129 | 21963 | 21834 | 2368 | 6201 | 10077 |
| #321 | D812 | 0.01 | 1649 | -55.25 | 129 | 19681 | 19552 | 1679 | 3574 | 9474 |
| #322 | D91 | 0.01 | 389 | -56.53 | 129 | 21576 | 21447 | 1765 | 4306 | 9733 |
| #323 | D92 | 0.01 | 1761 | -58.82 | 129 | 21791 | 21662 | 1808 | 3273 | 9776 |
| **Sound number (#)** | **Individual**  **sound**  **code** | **Acoustic parameters** | | | | | | | | |
|  |  | **Duration**  **(s)** | **Peak frequency (Hz)** | **Peak amplitude**  **(dB)** | **Min frequency (Hz)** | **Max frequency (Hz)** | **Bandwidth** | **Quartile 25** | **Quartile 50** | **Quartile 75** |
| #324 | D93 | 0.02 | 184 | -46.7 | 129 | 4177 | 4048 | 1119 | 2196 | 7192 |
| #325 | D94 | 0.01 | 216 | -47.55 | 129 | 5211 | 5081 | 1033 | 3919 | 8785 |
| #326 | D95 | 0.01 | 2111 | -49.56 | 129 | 21490 | 21360 | 1938 | 2928 | 9776 |
| #327 | D96 | 0.01 | 2894 | -51.1 | 129 | 21705 | 21576 | 2670 | 4522 | 10379 |
| #328 | D101 | 0.02 | 210 | -42.97 | 129 | 14211 | 14082 | 1248 | 2024 | 6632 |
| #329 | D102 | 0.01 | 1120 | -39.83 | 172 | 2153 | 1981 | 1119 | 1765 | 6546 |
| #330 | D103 | 0.01 | 181 | -52.72 | 129 | 6804 | 6675 | 990 | 3488 | 8914 |
| #331 | D104 | 0.01 | 6588 | -43.94 | 344 | 21963 | 21619 | 4694 | 6761 | 11455 |
| #332 | D105 | 0.01 | 1615 | -56.04 | 129 | 17872 | 17743 | 1378 | 3057 | 9130 |
| #333 | D106 | 0.02 | 393 | -50.2 | 129 | 7235 | 7106 | 818 | 2024 | 7149 |
| #334 | D107 | 0.02 | 171 | -53.05 | 129 | 7278 | 7149 | 1378 | 3273 | 9087 |
| #335 | D108 | 0.01 | 561 | -55.92 | 129 | 21705 | 21576 | 1938 | 5038 | 9733 |
| #336 | D109 | 0.01 | 6493 | -54.32 | 129 | 21834 | 21705 | 4478 | 6804 | 11240 |
| #337 | E11 | 0.01 | 248 | -35.5 | 129 | 2110 | 1981 | 344 | 1033 | 3617 |
| #338 | E12 | 0.01 | 281 | -47.29 | 129 | 9733 | 9603 | 947 | 3186 | 9216 |
| #339 | E13 | 0.01 | 491 | -34.51 | 129 | 2024 | 1894 | 516 | 861 | 2325 |
| #340 | E14 | 0.01 | 474 | -36.21 | 129 | 2196 | 2067 | 473 | 775 | 2024 |
| #341 | E21 | 0.01 | 342 | -38.99 | 129 | 3143 | 3014 | 646 | 1421 | 4737 |
| #342 | E22 | 0.01 | 192 | -47.78 | 129 | 5512 | 5383 | 689 | 1248 | 6330 |
| #343 | E23 | 0.01 | 433 | -39.06 | 129 | 2024 | 1894 | 516 | 904 | 4909 |
| #344 | E24 | 0.01 | 512 | -43.29 | 129 | 7106 | 6976 | 559 | 1550 | 6416 |
| #345 | E25 | 0.01 | 436 | -48.7 | 129 | 9388 | 9259 | 775 | 2885 | 8613 |
| #346 | E26 | 0.01 | 210 | -42.26 | 129 | 3531 | 3402 | 430 | 1292 | 5383 |
| #347 | E27 | 0.01 | 441 | -42.55 | 172 | 1981 | 1808 | 516 | 1076 | 4780 |
| #348 | E28 | 0.01 | 228 | -38.91 | 129 | 1808 | 1679 | 387 | 775 | 4909 |
| #349 | E29 | 0.01 | 214 | -36.65 | 129 | 1593 | 1464 | 344 | 732 | 2971 |
| #350 | E210 | 0.01 | 410 | -42.99 | 129 | 4823 | 4694 | 904 | 1679 | 6416 |
| #351 | E211 | 0.01 | 456 | -38.49 | 129 | 21705 | 21576 | 1205 | 2153 | 9345 |
| #352 | E212 | 0.01 | 371 | -37.5 | 129 | 2024 | 1894 | 646 | 1076 | 3574 |
| #353 | E213 | 0.01 | 385 | -31.43 | 129 | 3789 | 3660 | 559 | 1507 | 3703 |
| #354 | E214 | 0.01 | 515 | -33.01 | 215 | 2497 | 2282 | 732 | 1593 | 4392 |
| #355 | E31 | 0.01 | 548 | -35.43 | 129 | 2067 | 1938 | 559 | 904 | 4608 |
| #356 | E32 | 0.01 | 237 | -39.26 | 129 | 1335 | 1205 | 387 | 904 | 5943 |
| #357 | E33 | 0.01 | 392 | -42.83 | 129 | 3832 | 3703 | 559 | 1248 | 6072 |
| #358 | E34 | 0.01 | 203 | -45.21 | 129 | 6115 | 5986 | 430 | 1292 | 7062 |
| #359 | E41 | 0.01 | 270 | -43.12 | 172 | 1507 | 1335 | 344 | 1378 | 5943 |
| #360 | E42 | 0.01 | 172 | -50.39 | 129 | 6933 | 6804 | 1033 | 1851 | 7019 |
| #361 | E43 | 0.01 | 299 | -45.56 | 129 | 9646 | 9517 | 1248 | 1938 | 7019 |
| #362 | E44 | 0.01 | 562 | -49.85 | 129 | 21059 | 20930 | 861 | 2024 | 8354 |
| #363 | E45 | 0.02 | 175 | -46.27 | 129 | 5943 | 5814 | 1076 | 1765 | 6244 |
| #364 | E51 | 0.01 | 213 | -34.47 | 129 | 947 | 818 | 301 | 732 | 4780 |
| #365 | E52 | 0.01 | 240 | -34.31 | 129 | 2153 | 2024 | 387 | 904 | 2282 |
| **Sound number (#)** | **Individual**  **sound**  **code** | **Acoustic parameters** | | | | | | | | |
|  |  | **Duration**  **(s)** | **Peak frequency (Hz)** | **Peak amplitude**  **(dB)** | **Min frequency (Hz)** | **Max frequency (Hz)** | **Bandwidth** | **Quartile 25** | **Quartile 50** | **Quartile 75** |
| #366 | E53 | 0.01 | 336 | -34.69 | 129 | 14125 | 13996 | 732 | 1464 | 4306 |
| #367 | E54 | 0.01 | 1156 | -35.02 | 129 | 2153 | 2024 | 1033 | 1421 | 4091 |
| #368 | E55 | 0.02 | 443 | -39.74 | 129 | 1550 | 1421 | 473 | 689 | 6072 |
| #369 | E56 | 0.01 | 167 | -44.59 | 129 | 7062 | 6933 | 646 | 1292 | 6632 |
| #370 | E57 | 0.01 | 422 | -35.29 | 129 | 861 | 732 | 430 | 559 | 2153 |
| #371 | E58 | 0.01 | 206 | -37.95 | 129 | 1378 | 1248 | 301 | 602 | 3014 |
| #372 | E59 | 0.01 | 439 | -33.3 | 129 | 1248 | 1119 | 473 | 818 | 2584 |
| #373 | E510 | 0.01 | 448 | -39.31 | 172 | 1981 | 1808 | 559 | 1033 | 4737 |
| #374 | E61 | 0.01 | 765 | -51.16 | 129 | 9905 | 9776 | 990 | 2756 | 8957 |
| #375 | E62 | 0.01 | 781 | -57.05 | 129 | 21963 | 21834 | 1722 | 4737 | 9474 |
| #376 | E63 | 0.01 | 277 | -44.59 | 129 | 2153 | 2024 | 387 | 1679 | 6029 |
| #377 | E64 | 0.01 | 216 | -38.77 | 129 | 1938 | 1808 | 344 | 732 | 4952 |
| #378 | E65 | 0.03 | 278 | -36.19 | 129 | 1765 | 1636 | 344 | 861 | 3402 |
| #379 | E66 | 0.01 | 346 | -49.96 | 129 | 21447 | 21317 | 1248 | 2799 | 9000 |
| #380 | E67 | 0.02 | 195 | -30.17 | 129 | 818 | 689 | 258 | 387 | 1808 |
| #381 | E68 | 0.05 | 346 | -38.61 | 129 | 1679 | 1550 | 387 | 1292 | 6029 |
| #382 | E71 | 0.01 | 457 | -48.6 | 129 | 2110 | 1981 | 559 | 1808 | 7019 |
| #383 | E72 | 0.02 | 240 | -47.64 | 129 | 2928 | 2799 | 559 | 2153 | 7019 |
| #384 | E73 | 0.02 | 176 | -27.51 | 129 | 904 | 775 | 215 | 430 | 1593 |
| #385 | E74 | 0.01 | 247 | -43.32 | 129 | 2799 | 2670 | 775 | 1421 | 5641 |
| #386 | E75 | 0.01 | 256 | -42.09 | 129 | 1722 | 1593 | 430 | 1162 | 4909 |
| #387 | E76 | 0.01 | 235 | -32.51 | 129 | 4005 | 3876 | 646 | 1464 | 3316 |
| #388 | E77 | 0.01 | 340 | -33.56 | 129 | 1765 | 1636 | 430 | 1162 | 3402 |
| #389 | E81 | 0.01 | 270 | -52.48 | 129 | 10335 | 10206 | 646 | 3057 | 8268 |
| #390 | E82 | 0.01 | 343 | -48.3 | 129 | 5469 | 5340 | 473 | 2971 | 7062 |
| #391 | E83 | 0.01 | 438 | -46.99 | 129 | 9130 | 9000 | 646 | 1421 | 6976 |
| #392 | E84 | 0.01 | 367 | -40.93 | 129 | 3402 | 3273 | 473 | 1248 | 6546 |
| #393 | E85 | 0.01 | 506 | -46.58 | 129 | 9087 | 8957 | 646 | 1292 | 6460 |
| #394 | E86 | 0.01 | 440 | -49.96 | 129 | 9819 | 9689 | 646 | 1378 | 6589 |
| #395 | E87 | 0.01 | 398 | -44.81 | 172 | 6546 | 6373 | 602 | 1464 | 6460 |
| #396 | E88 | 0.01 | 241 | -40.62 | 129 | 2928 | 2799 | 430 | 818 | 3617 |
| #397 | E89 | 0.01 | 276 | -43.51 | 172 | 14427 | 14255 | 990 | 1894 | 6847 |
| #398 | E91 | 0.01 | 402 | -30.79 | 172 | 2067 | 1894 | 516 | 904 | 1981 |
| #399 | E92 | 0.01 | 210 | -34.24 | 129 | 1636 | 1507 | 301 | 1033 | 3445 |
| #400 | E93 | 0.01 | 192 | -35.62 | 129 | 4737 | 4608 | 732 | 1722 | 4177 |
| #401 | E94 | 0.01 | 308 | -39.7 | 129 | 1938 | 1808 | 473 | 1033 | 6330 |
| #402 | E95 | 0.01 | 234 | -32.92 | 129 | 3057 | 2928 | 344 | 1765 | 6330 |
| #403 | E96 | 0.01 | 223 | -34.93 | 129 | 1119 | 990 | 387 | 732 | 1981 |
| #404 | E97 | 0.02 | 240 | -49.28 | 172 | 6287 | 6115 | 516 | 2196 | 7019 |
| #405 | E98 | 0.01 | 332 | -36.74 | 129 | 1765 | 1636 | 344 | 775 | 3100 |
| #406 | E99 | 0.01 | 512 | -51.65 | 215 | 14125 | 13910 | 1162 | 2885 | 8957 |
| #407 | E910 | 0.01 | 262 | -43.71 | 172 | 2239 | 2067 | 732 | 1636 | 5340 |
| **Sound number (#)** | **Individual**  **sound**  **code** | **Acoustic parameters** | | | | | | | | |
|  |  | **Duration**  **(s)** | **Peak frequency (Hz)** | **Peak amplitude**  **(dB)** | **Min frequency (Hz)** | **Max frequency (Hz)** | **Bandwidth** | **Quartile 25** | **Quartile 50** | **Quartile 75** |
| #408 | E911 | 0.01 | 191 | -50.64 | 129 | 6029 | 5900 | 818 | 2454 | 7278 |
| #409 | E912 | 0.01 | 349 | -30.7 | 129 | 1636 | 1507 | 387 | 861 | 2024 |
| #410 | E913 | 0.01 | 234 | -33.26 | 129 | 1292 | 1162 | 301 | 775 | 2239 |
| #411 | E101 | 0.01 | 478 | -53.22 | 129 | 21920 | 21791 | 1292 | 2885 | 9087 |
| #412 | E102 | 0.01 | 413 | -51.16 | 129 | 6589 | 6460 | 775 | 1636 | 6632 |
| #413 | E103 | 0.02 | 509 | -56.79 | 129 | 21188 | 21059 | 1335 | 4435 | 9431 |
| #414 | E104 | 0.01 | 513 | -47.6 | 129 | 10981 | 10852 | 602 | 1248 | 6976 |
| #415 | E105 | 0.01 | 263 | -46.46 | 172 | 2627 | 2454 | 861 | 3746 | 9000 |
| #416 | E106 | 0.01 | 228 | -28.67 | 129 | 947 | 818 | 344 | 559 | 1033 |
| #417 | F11 | 0.01 | 874 | -50.57 | 172 | 14771 | 14599 | 1033 | 2067 | 8785 |
| #418 | F12 | 0.02 | 1110 | -45.53 | 129 | 8914 | 8785 | 990 | 1464 | 6847 |
| #419 | F13 | 0.02 | 467 | -31.11 | 129 | 2756 | 2627 | 516 | 1076 | 2627 |
| #420 | F14 | 0.01 | 258 | -43.59 | 129 | 9646 | 9517 | 732 | 2282 | 9043 |
| #421 | F15 | 0.01 | 199 | -20.87 | 129 | 602 | 473 | 258 | 473 | 2024 |
| #422 | F16 | 0.02 | 876 | -50.96 | 172 | 14771 | 14599 | 1033 | 2153 | 8957 |
| #423 | F17 | 0.01 | 194 | -17.85 | 129 | 1248 | 1119 | 258 | 602 | 1421 |
| #424 | F18 | 0.01 | 193 | -29.89 | 129 | 1335 | 1205 | 258 | 646 | 1765 |
| #425 | F19 | 0.01 | 221 | -25.8 | 129 | 990 | 861 | 301 | 559 | 1033 |
| #426 | F21 | 0.01 | 247 | -37.98 | 129 | 1248 | 1119 | 344 | 732 | 6287 |
| #427 | F22 | 0.02 | 220 | -46 | 129 | 1119 | 990 | 344 | 1593 | 6933 |
| #428 | F23 | 0.02 | 242 | -40.42 | 129 | 2153 | 2024 | 344 | 947 | 4608 |
| #429 | F24 | 0.02 | 195 | -36.54 | 129 | 689 | 559 | 258 | 430 | 3273 |
| #430 | F25 | 0.02 | 226 | -34.94 | 129 | 1162 | 1033 | 301 | 732 | 4651 |
| #431 | F26 | 0.01 | 293 | -43.15 | 129 | 1292 | 1162 | 344 | 947 | 5168 |
| #432 | F27 | 0.01 | 292 | -36.96 | 129 | 1292 | 1162 | 344 | 732 | 5555 |
| #433 | F28 | 0.02 | 1079 | -42.16 | 129 | 1981 | 1851 | 775 | 1119 | 5168 |
| #434 | F29 | 0.02 | 384 | -38.63 | 172 | 1981 | 1808 | 430 | 1076 | 6373 |
| #435 | F31 | 0.02 | 221 | -32.43 | 129 | 1981 | 1851 | 387 | 818 | 2067 |
| #436 | F32 | 0.01 | 250 | -36.02 | 129 | 1335 | 1205 | 344 | 689 | 4349 |
| #437 | F33 | 0.01 | 390 | -39.65 | 215 | 1765 | 1550 | 516 | 1205 | 6373 |
| #438 | F34 | 0.02 | 263 | -40.66 | 172 | 1421 | 1248 | 387 | 1248 | 5124 |
| #439 | F35 | 0.01 | 229 | -33.47 | 129 | 1378 | 1248 | 301 | 559 | 4694 |
| #440 | F36 | 0.01 | 256 | -37.13 | 129 | 516 | 387 | 301 | 602 | 4306 |
| #441 | F37 | 0.02 | 224 | -35.49 | 172 | 1335 | 1162 | 301 | 473 | 3057 |
| #442 | F38 | 0.03 | 247 | -45.11 | 172 | 6761 | 6589 | 1119 | 1851 | 7192 |
| #443 | F39 | 0.03 | 237 | -39.12 | 172 | 387 | 215 | 301 | 1335 | 6546 |
| #444 | F310 | 0.01 | 216 | -22.97 | 129 | 1076 | 947 | 301 | 559 | 1076 |
| #445 | F311 | 0.01 | 332 | -41.92 | 129 | 2024 | 1894 | 430 | 990 | 6029 |
| #446 | F312 | 0.03 | 221 | -42.73 | 172 | 1421 | 1248 | 387 | 1894 | 6460 |
| #447 | F313 | 0.02 | 245 | -48.7 | 129 | 13652 | 13522 | 1205 | 2670 | 8914 |
| #448 | F41 | 0.01 | 638 | -50.77 | 172 | 6503 | 6330 | 775 | 1550 | 6503 |
| #449 | F42 | 0.01 | 229 | -36.58 | 172 | 1550 | 1378 | 301 | 1378 | 4995 |
| **Sound number (#)** | **Individual**  **sound**  **code** | **Acoustic parameters** | | | | | | | | |
|  |  | **Duration**  **(s)** | **Peak frequency (Hz)** | **Peak amplitude**  **(dB)** | **Min frequency (Hz)** | **Max frequency (Hz)** | **Bandwidth** | **Quartile 25** | **Quartile 50** | **Quartile 75** |
| #450 | F43 | 0.01 | 257 | -40.82 | 172 | 1033 | 861 | 344 | 818 | 4694 |
| #451 | F44 | 0.01 | 229 | -30.86 | 129 | 2282 | 2153 | 732 | 1205 | 2627 |
| #452 | F45 | 0.03 | 188 | -34.06 | 129 | 1335 | 1205 | 301 | 516 | 3230 |
| #453 | F46 | 0.01 | 354 | -30.31 | 129 | 861 | 732 | 344 | 473 | 1593 |
| #454 | F51 | 0.01 | 231 | -30.78 | 129 | 1507 | 1378 | 344 | 559 | 1378 |
| #455 | F52 | 0.02 | 249 | -29.67 | 129 | 1248 | 1119 | 301 | 602 | 3531 |
| #456 | F53 | 0.01 | 379 | -32.29 | 129 | 1335 | 1205 | 387 | 904 | 1981 |
| #457 | F54 | 0.01 | 223 | -24.28 | 129 | 861 | 732 | 301 | 430 | 818 |
| #458 | F55 | 0.01 | 228 | -28.89 | 129 | 1981 | 1851 | 430 | 947 | 1894 |
| #459 | F56 | 0.02 | 365 | -31.78 | 129 | 1335 | 1205 | 344 | 646 | 1248 |
| #460 | F57 | 0.01 | 368 | -29.11 | 129 | 2239 | 2110 | 473 | 1033 | 2196 |
| #461 | F61 | 0.02 | 269 | -41.51 | 129 | 7278 | 7149 | 430 | 732 | 6503 |
| #462 | F62 | 0.02 | 257 | -35.21 | 129 | 1335 | 1205 | 387 | 775 | 3617 |
| #463 | F63 | 0.01 | 204 | -29.23 | 129 | 1421 | 1292 | 301 | 516 | 1765 |
| #464 | F64 | 0.01 | 253 | -37.62 | 172 | 1679 | 1507 | 430 | 947 | 4263 |
| #465 | F65 | 0.01 | 269 | -39.26 | 129 | 1378 | 1248 | 387 | 775 | 5340 |
| #466 | F66 | 0.03 | 175 | -36.17 | 129 | 2627 | 2497 | 559 | 1335 | 3488 |
| #467 | F67 | 0.03 | 196 | -39.02 | 129 | 1464 | 1335 | 258 | 1292 | 5641 |
| #468 | F68 | 0.01 | 244 | -43.91 | 172 | 1464 | 1292 | 344 | 1335 | 5469 |
| #469 | F69 | 0.01 | 265 | -35.73 | 129 | 1378 | 1248 | 344 | 473 | 3531 |
| #470 | F610 | 0.01 | 220 | -33.67 | 129 | 1421 | 1292 | 344 | 947 | 2239 |
| #471 | F611 | 0.01 | 240 | -32.22 | 129 | 1162 | 1033 | 301 | 775 | 2842 |
| #472 | F612 | 0.01 | 1111 | -52.8 | 129 | 7149 | 7019 | 1076 | 2885 | 8096 |
| #473 | F613 | 0.02 | 181 | -36.21 | 129 | 818 | 689 | 258 | 732 | 5340 |
| #474 | F614 | 0.01 | 553 | -40.52 | 172 | 14298 | 14125 | 904 | 1808 | 4866 |
| #475 | F615 | 0.03 | 245 | -31.43 | 172 | 1335 | 1162 | 301 | 732 | 2885 |
| #476 | F616 | 0.01 | 1240 | -40.05 | 129 | 5081 | 4952 | 1119 | 1550 | 5857 |
| #477 | F71 | 0.01 | 592 | -37.55 | 172 | 2971 | 2799 | 602 | 1076 | 3531 |
| #478 | F72 | 0.01 | 264 | -35.16 | 129 | 1292 | 1162 | 387 | 602 | 2842 |
| #479 | F73 | 0.01 | 215 | -35.64 | 129 | 1938 | 1808 | 301 | 473 | 1894 |
| #480 | F74 | 0.02 | 198 | -36.83 | 129 | 1981 | 1851 | 387 | 732 | 3962 |
| #481 | F75 | 0.01 | 344 | -24.63 | 172 | 1248 | 1076 | 387 | 646 | 1248 |
| #482 | F76 | 0.02 | 188 | -34.56 | 129 | 1292 | 1162 | 387 | 646 | 2110 |
| #483 | F77 | 0.02 | 373 | -25.87 | 129 | 1981 | 1851 | 430 | 818 | 1292 |
| #484 | F78 | 0.02 | 198 | -35.02 | 129 | 1765 | 1636 | 301 | 947 | 4995 |
| #485 | F79 | 0.02 | 286 | -40.8 | 129 | 2282 | 2153 | 473 | 1162 | 6244 |
| #486 | F710 | 0.01 | 645 | -45.81 | 129 | 14298 | 14168 | 775 | 1636 | 6158 |
| #487 | F711 | 0.01 | 1087 | -38.86 | 129 | 3617 | 3488 | 1162 | 1808 | 7321 |
| #488 | F81 | 0.02 | 231 | -32.52 | 129 | 1378 | 1248 | 344 | 904 | 2670 |
| #489 | F82 | 0.01 | 1161 | -46 | 129 | 9733 | 9603 | 1119 | 1981 | 8871 |
| #490 | F83 | 0.01 | 360 | -48.2 | 129 | 4005 | 3876 | 1119 | 1851 | 6890 |
| #491 | F84 | 0.01 | 329 | -33.05 | 129 | 1894 | 1765 | 387 | 904 | 2756 |
| **Sound number (#)** | **Individual**  **sound**  **code** | **Acoustic parameters** | | | | | | | | |
|  |  | **Duration**  **(s)** | **Peak frequency (Hz)** | **Peak amplitude**  **(dB)** | **Min frequency (Hz)** | **Max frequency (Hz)** | **Bandwidth** | **Quartile 25** | **Quartile 50** | **Quartile 75** |
| #492 | F85 | 0.01 | 197 | -36.07 | 129 | 818 | 689 | 301 | 646 | 6287 |
| #493 | F86 | 0.01 | 229 | -20.28 | 129 | 516 | 387 | 258 | 344 | 473 |
| #494 | F87 | 0.01 | 333 | -48.06 | 129 | 14255 | 14125 | 904 | 2670 | 9259 |
| #495 | F88 | 0.02 | 372 | -27.59 | 129 | 1248 | 1119 | 387 | 473 | 1248 |
| #496 | F89 | 0.01 | 219 | -47.2 | 129 | 9431 | 9302 | 732 | 1808 | 6761 |
| #497 | F810 | 0.03 | 268 | -25.78 | 129 | 1808 | 1679 | 301 | 387 | 1808 |
| #498 | F91 | 0.02 | 195 | -45.04 | 129 | 1335 | 1205 | 301 | 1205 | 6158 |
| #499 | F92 | 0.03 | 195 | -43.82 | 129 | 4522 | 4392 | 473 | 947 | 6287 |
| #500 | F93 | 0.01 | 229 | -36.1 | 129 | 646 | 516 | 301 | 516 | 4177 |
| #501 | F94 | 0.02 | 223 | -38.63 | 129 | 1205 | 1076 | 344 | 775 | 6546 |
| #502 | F95 | 0.01 | 162 | -36.28 | 129 | 602 | 473 | 215 | 559 | 3574 |
| #503 | F96 | 0.01 | 230 | -29.66 | 129 | 732 | 602 | 301 | 430 | 1162 |
| #504 | F97 | 0.01 | 224 | -29.77 | 129 | 775 | 646 | 258 | 387 | 1808 |
| #505 | F98 | 0.01 | 233 | -27.84 | 129 | 818 | 689 | 301 | 430 | 1205 |
| #506 | F99 | 0.02 | 405 | -41.36 | 172 | 1938 | 1765 | 516 | 818 | 5124 |
| #507 | F910 | 0.01 | 219 | -31.22 | 129 | 1335 | 1205 | 301 | 732 | 2067 |
| #508 | F911 | 0.02 | 424 | -36.26 | 129 | 1119 | 990 | 430 | 559 | 4177 |
| #509 | F912 | 0.01 | 243 | -30.88 | 129 | 1507 | 1378 | 344 | 775 | 1507 |
| #510 | F913 | 0.01 | 332 | -40.07 | 86 | 2325 | 2239 | 473 | 1205 | 5168 |
| #511 | F101 | 0.02 | 173 | -35.92 | 129 | 861 | 732 | 344 | 689 | 4823 |
| #512 | F102 | 0.01 | 454 | -43.29 | 129 | 4995 | 4866 | 516 | 1464 | 4909 |
| #513 | F103 | 0.01 | 378 | -34.54 | 129 | 3143 | 3014 | 430 | 861 | 1851 |
| #514 | F104 | 0.01 | 215 | -32.93 | 129 | 516 | 387 | 258 | 430 | 1894 |
| #515 | F105 | 0.02 | 284 | -37.43 | 129 | 1981 | 1851 | 430 | 1421 | 6373 |
| #516 | F106 | 0.01 | 238 | -27.08 | 129 | 1162 | 1033 | 301 | 430 | 1119 |

b. Wild recorded sounds (Pachón cave)

|  |  | **Acoustic parameters** | | | | | | | | | |
| --- | --- | --- | --- | --- | --- | --- | --- | --- | --- | --- | --- |
| **Sound number**  **(#)** | **Sound code** | **Duration (s)** | **Peak frequency (Hz)** | **Peak amplitude (dB)** | **Min frequency (Hz)** | **Max frequency (Hz)** | **Bandwidth** | **Quartile 25** | | **Quartile 50** | **Quartile 100** |
| #1 | X1 | 0.034 | 100 | -33.75 | 100 | 800 | 600 | 300 | 500 | | 5500 |
| #2 | X2 | 0.008 | 100 | -11.01 | 100 | 800 | 600 | 300 | 500 | | 600 |
| #3 | X3 | 0.014 | 100 | -40.02 | 100 | 600 | 500 | 500 | 3600 | | 12000 |
| #4 | X4 | 0.008 | 300 | -42.5 | 100 | 800 | 600 | 500 | 3400 | | 11000 |
| #5 | X5 | 0.005 | 300 | -28.31 | 100 | 800 | 600 | 300 | 500 | | 800 |
| #6 | X6 | 0.034 | 100 | -30.75 | 100 | 600 | 500 | 300 | 500 | | 5800 |
| #7 | X7 | 0.049 | 100 | -36.44 | 100 | 600 | 500 | 300 | 2400 | | 11100 |
| #8 | X8 | 0.011 | 100 | -43.8 | 100 | 800 | 600 | 500 | 6500 | | 12700 |
| #9 | X9 | 0.014 | 100 | -40.17 | 100 | 600 | 500 | 300 | 3400 | | 11100 |
| #10 | X10 | 0.011 | 100 | -45.39 | 100 | 800 | 600 | 800 | 6500 | | 13400 |
| #11 | X11 | 0.02 | 100 | -42.28 | 100 | 600 | 500 | 500 | 5100 | | 11700 |
|  |  | **Acoustic parameters** | | | | | | | | | |
| **Sound number**  **(#)** | **Sound code** | **Duration (s)** | **Peak frequency (Hz)** | **Peak amplitude (dB)** | **Min frequency (Hz)** | **Max frequency (Hz)** | **Bandwidth** | **Quartile 25** | | **Quartile 50** | **Quartile 100** |
| #12 | X12 | 0.02 | 100 | -37.1 | 100 | 800 | 600 | 300 | 1500 | | 9600 |
| #13 | X13 | 0.005 | 100 | -37.48 | 100 | 600 | 500 | 300 | 1800 | | 10300 |
| #14 | X14 | 0.005 | 100 | -40.24 | 100 | 800 | 600 | 500 | 3600 | | 12200 |
| #15 | X15 | 0.031 | 100 | -40.54 | 100 | 800 | 600 | 500 | 3400 | | 11300 |
| #16 | X16 | 0.026 | 100 | -41.62 | 100 | 600 | 500 | 500 | 5600 | | 12000 |
| #17 | X17 | 0.037 | 100 | -34.7 | 100 | 600 | 500 | 300 | 500 | | 8700 |
| #18 | X18 | 0.014 | 100 | -34.26 | 100 | 600 | 500 | 300 | 500 | | 8700 |
| #19 | X19 | 0.049 | 100 | -40.74 | 100 | 600 | 500 | 500 | 5600 | | 12900 |
| #20 | X20 | 0.014 | 100 | -41.38 | 100 | 600 | 500 | 500 | 3900 | | 11000 |
| #21 | X21 | 0.026 | 100 | -41.65 | 100 | 600 | 500 | 500 | 5800 | | 14200 |
| #22 | X22 | 0.037 | 100 | -31.57 | 100 | 600 | 500 | 300 | 500 | | 6200 |
| #23 | X23 | 0.023 | 100 | -36.42 | 100 | 800 | 600 | 300 | 1000 | | 9400 |
| #24 | X24 | 0.055 | 100 | -43.12 | 100 | 600 | 500 | 500 | 6200 | | 12400 |
| #25 | X25 | 0.034 | 100 | -42.65 | 100 | 600 | 500 | 500 | 4900 | | 13200 |
| #26 | X26 | 0.031 | 100 | -40.44 | 100 | 800 | 600 | 500 | 3600 | | 10500 |
| #27 | X27 | 0.02 | 100 | -35.27 | 100 | 600 | 500 | 300 | 1200 | | 10500 |
| #28 | X28 | 0.052 | 100 | -41.38 | 100 | 600 | 500 | 500 | 5300 | | 12200 |
| #29 | X29 | 0.026 | 100 | -32.98 | 100 | 600 | 500 | 300 | 500 | | 7500 |
| #30 | X30 | 0.069 | 100 | -40.48 | 100 | 600 | 500 | 500 | 4800 | | 12000 |
| #31 | X31 | 0.023 | 100 | -39.43 | 100 | 800 | 600 | 500 | 2900 | | 10100 |
| #32 | X32 | 0.037 | 100 | -40.36 | 100 | 800 | 600 | 500 | 2000 | | 11100 |
| #33 | X33 | 0.034 | 100 | -33.41 | 100 | 600 | 500 | 300 | 1000 | | 9800 |
| #34 | X34 | 0.04 | 100 | -41.55 | 100 | 600 | 500 | 500 | 5800 | | 12500 |
| #35 | X35 | 0.052 | 100 | -41.22 | 100 | 800 | 600 | 500 | 4100 | | 11100 |
| #36 | X36 | 0.04 | 100 | -37.88 | 100 | 600 | 500 | 300 | 3600 | | 11500 |
| #37 | X37 | 0.037 | 100 | -29.17 | 100 | 800 | 600 | 300 | 500 | | 3200 |
| #38 | X38 | 0.04 | 100 | -37.69 | 100 | 600 | 500 | 300 | 2000 | | 9900 |
| #39 | X39 | 0.037 | 100 | -35.27 | 100 | 600 | 500 | 300 | 600 | | 10500 |
| #40 | X40 | 0.04 | 100 | -38.52 | 100 | 600 | 500 | 300 | 3900 | | 11800 |
| #41 | X41 | 0.008 | 100 | -37.59 | 100 | 600 | 500 | 300 | 3100 | | 11000 |
| #42 | X42 | 0.014 | 100 | -38.35 | 100 | 800 | 600 | 500 | 600 | | 8000 |
| #43 | X43 | 0.011 | 100 | -28.45 | 100 | 600 | 500 | 300 | 500 | | 5600 |
| #44 | X44 | 0.04 | 100 | -28.54 | 100 | 600 | 500 | 300 | 500 | | 6200 |
| #45 | X45 | 0.055 | 100 | -35.01 | 100 | 600 | 500 | 300 | 500 | | 8000 |
| #46 | Y1 | 0.002 | 2700 | -51.3 | 600 | 6200 | 5500 | 2200 | 3100 | | 4800 |
| #47 | Y2 | 0.005 | 3600 | -51.44 | 1700 | 11500 | 9800 | 3400 | 4400 | | 7000 |
| #48 | Y3 | 0.002 | 1700 | -49.28 | 1000 | 6700 | 5600 | 2000 | 2700 | | 4900 |
| #49 | Y4 | 0.002 | 1700 | -49.39 | 1000 | 3100 | 2000 | 1700 | 2000 | | 3700 |
| #50 | Y5 | 0.002 | 3200 | -55.69 | 500 | 17500 | 17000 | 3400 | 5500 | | 12700 |
| #51 | Y6 | 0.002 | 3100 | -53.75 | 100 | 17200 | 17000 | 3200 | 4600 | | 9900 |
| #52 | Y7 | 0.002 | 12000 | -57.46 | 100 | 21700 | 21500 | 9600 | 11800 | | 14400 |
| #53 | Y8 | 0.002 | 6500 | -64.37 | 100 | 21700 | 21500 | 4800 | 7400 | | 12500 |
| #54 | Y9 | 0.002 | 1800 | -54.42 | 800 | 6700 | 5800 | 1800 | 2900 | | 5300 |
| #55 | Y10 | 0.002 | 1800 | -55.92 | 100 | 14400 | 14200 | 1800 | 2500 | | 9300 |
| #56 | Y11 | 0.002 | 1700 | -54.42 | 100 | 6300 | 6200 | 2000 | 3400 | | 4800 |
|  |  | **Acoustic parameters** | | | | | | | | | |
| **Sound number**  **(#)** | **Sound code** | **Duration (s)** | **Peak frequency (Hz)** | **Peak amplitude (dB)** | **Min frequency (Hz)** | **Max frequency (Hz)** | **Bandwidth** | **Quartile 25** | | **Quartile 50** | **Quartile 100** |
| #57 | Y12 | 0.002 | 2700 | -54.93 | 100 | 6000 | 5800 | 2700 | 3700 | | 9400 |
| #58 | Y13 | 0.002 | 1700 | -47.78 | 1200 | 7200 | 6000 | 2000 | 3100 | | 4900 |
| #59 | Y14 | 0.008 | 3100 | -53.94 | 100 | 6500 | 6300 | 3100 | 3600 | | 6200 |
| #60 | Y15 | 0.002 | 2700 | -54.22 | 600 | 5300 | 4600 | 2700 | 3700 | | 7200 |
| #61 | Y16 | 0.002 | 2500 | -63.21 | 100 | 19100 | 18900 | 2500 | 4400 | | 12000 |
| #62 | Y17 | 0.002 | 2700 | -56.79 | 100 | 21100 | 21000 | 2700 | 3600 | | 7700 |
| #63 | Y18 | 0.002 | 2900 | -47.03 | 1300 | 6200 | 4800 | 2200 | 3100 | | 4400 |
| #64 | Y19 | 0.002 | 1800 | -37.86 | 1300 | 6300 | 4900 | 2200 | 2900 | | 3900 |
| #65 | Y20 | 0.002 | 2500 | -54.22 | 100 | 6200 | 6000 | 2700 | 3200 | | 6200 |
| #66 | Y21 | 0.002 | 3400 | -58.04 | 100 | 19600 | 19400 | 2700 | 3700 | | 7500 |
| #67 | Y22 | 0.005 | 1300 | -49.01 | 1000 | 5800 | 4800 | 1700 | 2400 | | 3700 |
| #68 | Y23 | 0.002 | 2200 | -50.77 | 100 | 10300 | 10100 | 2500 | 3700 | | 5500 |
| #69 | Y24 | 0.002 | 1700 | -59.16 | 100 | 20600 | 20400 | 2000 | 3200 | | 8900 |
| #70 | Y25 | 0.002 | 2700 | -50.2 | 1200 | 6800 | 5600 | 2400 | 3100 | | 4800 |
| #71 | Y26 | 0.002 | 2700 | -51.73 | 1200 | 6800 | 5600 | 2400 | 3100 | | 4900 |
| #72 | Y27 | 0.002 | 1700 | -51.51 | 100 | 8200 | 8000 | 2900 | 4100 | | 6500 |
| #73 | Y28 | 0.002 | 2000 | -61.71 | 100 | 21500 | 21300 | 2000 | 3200 | | 9300 |
| #74 | Y29 | 0.002 | 2700 | -54.42 | 100 | 5300 | 5100 | 2900 | 3600 | | 7200 |
| #75 | Y30 | 0.002 | 2700 | -52.89 | 100 | 7200 | 7000 | 2200 | 3200 | | 5300 |
| #76 | Y31 | 0.002 | 1800 | -58.04 | 300 | 17000 | 16700 | 2500 | 4600 | | 8000 |
| #77 | Y32 | 0.002 | 2500 | -57.89 | 100 | 18000 | 17900 | 2400 | 3400 | | 5500 |
| #78 | Y33 | 0.002 | 1800 | -56.41 | 300 | 5100 | 4800 | 1800 | 2900 | | 4900 |
| #79 | Y34 | 0.002 | 2500 | -60.64 | 100 | 20300 | 20100 | 2900 | 4400 | | 11100 |
| #80 | Y35 | 0.002 | 3100 | -56.53 | 600 | 15100 | 14400 | 2700 | 3400 | | 8200 |
| #81 | Y36 | 0.002 | 1800 | -52.89 | 100 | 5300 | 5100 | 1800 | 2400 | | 4300 |
| #82 | Y37 | 0.002 | 2500 | -51.03 | 600 | 4100 | 3400 | 2500 | 2900 | | 5100 |
| #83 | Y38 | 0.002 | 3100 | -50.39 | 800 | 4400 | 3600 | 2200 | 3100 | | 3700 |
| #84 | Y39 | 0.002 | 2500 | -46.58 | 1300 | 8400 | 7000 | 2900 | 4300 | | 5800 |
| #85 | Y40 | 0.002 | 1300 | -40.91 | 800 | 2900 | 2000 | 1500 | 1700 | | 2400 |
| #86 | Y41 | 0.002 | 2000 | -41.53 | 1300 | 3900 | 2500 | 2000 | 2400 | | 2700 |
| #87 | Y42 | 0.002 | 1800 | -53.48 | 100 | 5600 | 5500 | 1800 | 2500 | | 6700 |
| #88 | Y43 | 0.002 | 1800 | -51.65 | 1000 | 4600 | 3600 | 2000 | 2900 | | 4100 |
| #89 | Y44 | 0.002 | 1700 | -46.99 | 800 | 4900 | 4100 | 1800 | 2400 | | 3200 |
