# Supplementary material for: Evolution of acoustic communication in blind cavefish
Source: Nat Commun. 2019 Sep 17;10:4231. doi: 10.1038/s41467-019-12078-9 (PMC6748933; doi:10.1038/s41467-019-12078-9)
Supplement: Supplementary file 25 — Supplementary Data 2 [file 41467_2019_12078_MOESM25_ESM.doc]

**Supplementary data 2**: Acoustic parameters (column 3 to 7) of 186 complex sounds recorded in the lab (n=12 SF, n=9 Pachón CF), used for temporal description and pulse rate analysis. Number of sound (#) is in column 1. Individual sound code (column 2) indicates morph and sound categories with a capital letter A, B (light grey) = Serial clocs for SF and CF respectively, C, D (light green) = Serial clicks for SF and CF respectively. **Min** = minimum, **Max** = maximum, **s** = second, **Hz** = hertz, **dB** = decibel.

|  |  | **Acoustic parameters** | | | | |
| --- | --- | --- | --- | --- | --- | --- |
| **Sound number (#)** | **Code** | **Duration (s)** | **Pulse number** | **Pulse duration (s)** | **Interpulse duration (s)** | **Pulse rate** |
| #1 | A11 | 0.35 | 4.00 | 0.04 | 0.06 | 11.56 |
| #2 | A12 | 0.39 | 3.00 | 0.05 | 0.12 | 7.64 |
| #3 | A13 | 0.16 | 2.00 | 0.07 | 0.02 | 12.38 |
| #4 | A14 | 0.43 | 4.00 | 0.04 | 0.09 | 9.38 |
| #5 | A21 | 0.15 | 3.00 | 0.04 | 0.01 | 20.03 |
| #6 | A22 | 0.21 | 2.00 | 0.08 | 0.05 | 9.61 |
| #7 | A23 | 0.18 | 2.00 | 0.06 | 0.06 | 10.89 |
| #8 | A24 | 0.20 | 2.00 | 0.09 | 0.02 | 10.21 |
| #9 | A25 | 0.65 | 4.00 | 0.04 | 0.16 | 6.20 |
| #10 | A26 | 0.59 | 5.00 | 0.08 | 0.05 | 8.53 |
| #11 | A27 | 0.39 | 3.00 | 0.09 | 0.06 | 7.66 |
| #12 | A28 | 0.50 | 4.00 | 0.08 | 0.05 | 8.04 |
| #13 | A29 | 0.39 | 3.00 | 0.10 | 0.05 | 7.64 |
| #14 | A31 | 0.44 | 2.00 | 0.07 | 0.29 | 4.56 |
| #15 | A41 | 0.25 | 2.00 | 0.08 | 0.09 | 7.90 |
| #16 | A42 | 0.32 | 3.00 | 0.07 | 0.06 | 9.25 |
| #17 | A43 | 0.25 | 2.00 | 0.08 | 0.09 | 7.91 |
| #18 | A44 | 0.32 | 2.00 | 0.11 | 0.09 | 6.22 |
| #19 | A51 | 0.37 | 2.00 | 0.12 | 0.14 | 5.42 |
| #20 | A61 | 0.32 | 2.00 | 0.08 | 0.16 | 6.22 |
| #21 | A62 | 0.26 | 2.00 | 0.10 | 0.07 | 7.58 |
| #22 | A63 | 0.20 | 2.00 | 0.09 | 0.02 | 10.18 |
| #23 | A64 | 0.25 | 2.00 | 0.08 | 0.10 | 7.87 |
| #24 | A71 | 0.26 | 2.00 | 0.06 | 0.15 | 7.57 |
| #25 | A81 | 0.18 | 2.00 | 0.08 | 0.02 | 10.84 |
| #26 | A91 | 0.28 | 2.00 | 0.09 | 0.10 | 7.18 |
| #27 | A92 | 0.82 | 4.00 | 0.05 | 0.21 | 4.86 |
| #28 | A93 | 0.20 | 2.00 | 0.06 | 0.08 | 10.14 |
| #29 | A94 | 0.37 | 2.00 | 0.07 | 0.23 | 5.40 |
| #30 | A95 | 0.90 | 5.00 | 0.08 | 0.13 | 5.56 |
| #31 | A96 | 0.46 | 3.00 | 0.10 | 0.08 | 6.47 |
| #32 | A97 | 0.95 | 4.00 | 0.05 | 0.25 | 4.22 |
| #33 | A98 | 0.28 | 2.00 | 0.11 | 0.06 | 7.27 |
| #34 | A99 | 0.24 | 2.00 | 0.06 | 0.12 | 8.21 |
| #35 | A910 | 0.29 | 2.00 | 0.09 | 0.11 | 6.96 |
| #36 | A911 | 0.30 | 3.00 | 0.05 | 0.07 | 10.04 |
| #37 | A101 | 0.36 | 2.00 | 0.12 | 0.12 | 5.60 |
| #38 | A102 | 0.60 | 3.00 | 0.09 | 0.17 | 4.98 |
| #39 | A103 | 0.44 | 2.00 | 0.07 | 0.29 | 4.57 |
| #40 | A104 | 0.27 | 2.00 | 0.09 | 0.08 | 7.51 |
| #41 | A105 | 0.26 | 2.00 | 0.06 | 0.15 | 7.56 |
| #42 | A106 | 1.57 | 6.00 | 0.06 | 0.24 | 3.82 |
| #43 | A107 | 0.83 | 5.00 | 0.07 | 0.15 | 6.00 |
| #44 | A108 | 0.19 | 2.00 | 0.08 | 0.02 | 10.80 |
| #45 | A109 | 0.72 | 4.00 | 0.11 | 0.13 | 5.57 |
| #46 | A1010 | 0.78 | 4.00 | 0.07 | 0.16 | 5.16 |
| #47 | A1011 | 0.86 | 5.00 | 0.06 | 0.14 | 5.84 |
| #48 | A1012 | 0.75 | 4.00 | 0.03 | 0.20 | 5.32 |
|  |  | **Acoustic parameters** | | | | |
| **Sound number (#)** | **Code** | **Duration (s)** | **Pulse number** | **Pulse duration (s)** | **Interpulse duration (s)** | **Pulse rate** |
| #49 | A1013 | 0.77 | 4.00 | 0.09 | 0.14 | 5.17 |
| #50 | A1014 | 0.26 | 2.00 | 0.12 | 0.02 | 7.56 |
| #51 | A1015 | 0.84 | 4.00 | 0.06 | 0.20 | 4.75 |
| #52 | A1016 | 0.26 | 2.00 | 0.10 | 0.07 | 7.56 |
| #53 | A1017 | 0.38 | 4.00 | 0.04 | 0.07 | 10.56 |
| #54 | A1018 | 0.25 | 2.00 | 0.08 | 0.09 | 7.90 |
| #55 | A111 | 0.38 | 2.00 | 0.06 | 0.25 | 5.27 |
| #56 | A112 | 0.40 | 2.00 | 0.10 | 0.21 | 4.95 |
| #57 | A113 | 0.74 | 4.00 | 0.08 | 0.14 | 5.39 |
| #58 | A114 | 0.42 | 2.00 | 0.10 | 0.22 | 4.79 |
| #59 | A115 | 0.78 | 5.00 | 0.07 | 0.11 | 6.43 |
| #60 | A116 | 0.40 | 2.00 | 0.12 | 0.16 | 4.96 |
| #61 | A117 | 0.41 | 3.00 | 0.09 | 0.07 | 7.26 |
| #62 | A118 | 0.48 | 2.00 | 0.15 | 0.18 | 4.15 |
| #63 | A119 | 0.66 | 3.00 | 0.08 | 0.21 | 4.57 |
| #64 | A1110 | 0.73 | 4.00 | 0.11 | 0.10 | 5.49 |
| #65 | A1111 | 0.45 | 2.00 | 0.09 | 0.28 | 4.44 |
| #66 | A1112 | 0.76 | 3.00 | 0.07 | 0.28 | 3.95 |
| #67 | A1113 | 0.53 | 3.00 | 0.10 | 0.12 | 5.67 |
| #68 | A121 | 0.45 | 4.00 | 0.06 | 0.07 | 8.90 |
| #69 | A122 | 0.18 | 2.00 | 0.09 | 0.01 | 10.83 |
| #70 | A123 | 0.48 | 4.00 | 0.08 | 0.05 | 8.26 |
| #71 | A124 | 0.22 | 3.00 | 0.07 | 0.01 | 13.71 |
| #72 | B11 | 0.32 | 2.00 | 0.08 | 0.16 | 6.20 |
| #73 | B12 | 0.33 | 2.00 | 0.11 | 0.12 | 5.99 |
| #74 | B13 | 0.23 | 2.00 | 0.10 | 0.03 | 8.63 |
| #75 | B21 | 0.20 | 2.00 | 0.08 | 0.03 | 10.25 |
| #76 | B22 | 0.41 | 3.00 | 0.11 | 0.05 | 7.24 |
| #77 | B23 | 0.45 | 3.00 | 0.07 | 0.12 | 6.70 |
| #78 | B24 | 0.64 | 3.00 | 0.10 | 0.17 | 4.71 |
| #79 | B25 | 0.21 | 2.00 | 0.07 | 0.06 | 9.69 |
| #80 | B26 | 0.24 | 2.00 | 0.07 | 0.10 | 8.21 |
| #81 | B27 | 0.36 | 3.00 | 0.10 | 0.03 | 8.40 |
| #82 | B28 | 0.39 | 3.00 | 0.09 | 0.06 | 7.62 |
| #83 | B29 | 0.29 | 2.00 | 0.08 | 0.13 | 6.94 |
| #84 | B210 | 0.28 | 3.00 | 0.05 | 0.06 | 10.85 |
| #85 | B211 | 0.51 | 4.00 | 0.11 | 0.03 | 7.87 |
| #86 | B212 | 0.32 | 3.00 | 0.08 | 0.03 | 9.32 |
| #87 | B213 | 0.61 | 3.00 | 0.07 | 0.20 | 4.89 |
| #88 | B214 | 0.56 | 4.00 | 0.07 | 0.10 | 7.09 |
| #89 | B215 | 0.41 | 4.00 | 0.04 | 0.08 | 9.64 |
| #90 | B31 | 0.22 | 2.00 | 0.10 | 0.01 | 9.08 |
| #91 | B32 | 0.30 | 2.00 | 0.12 | 0.07 | 6.68 |
| #92 | B33 | 0.24 | 2.00 | 0.08 | 0.08 | 8.29 |
| #93 | B41 | 0.42 | 2.00 | 0.09 | 0.24 | 4.80 |
| #94 | B42 | 0.25 | 2.00 | 0.10 | 0.06 | 7.91 |
|  |  | **Acoustic parameters** | | | | |
| **Sound number (#)** | **Code** | **Duration (s)** | **Pulse number** | **Pulse duration (s)** | **Interpulse duration (s)** | **Pulse rate** |
| #95 | B43 | 0.44 | 2.00 | 0.12 | 0.21 | 4.57 |
| #96 | B44 | 0.29 | 2.00 | 0.08 | 0.13 | 6.96 |
| #97 | B45 | 0.79 | 4.00 | 0.07 | 0.17 | 5.08 |
| #98 | B46 | 0.39 | 4.00 | 0.07 | 0.04 | 10.22 |
| #99 | B47 | 0.31 | 4.00 | 0.06 | 0.02 | 12.87 |
| #100 | B48 | 0.48 | 4.00 | 0.07 | 0.07 | 8.25 |
| #101 | B51 | 0.23 | 2.00 | 0.10 | 0.02 | 8.67 |
| #102 | B52 | 0.24 | 2.00 | 0.09 | 0.06 | 8.30 |
| #103 | B53 | 0.53 | 4.00 | 0.08 | 0.07 | 7.55 |
| #104 | B54 | 0.27 | 2.00 | 0.10 | 0.07 | 7.52 |
| #105 | B61 | 0.65 | 4.00 | 0.07 | 0.12 | 6.18 |
| #106 | B62 | 0.46 | 4.00 | 0.07 | 0.06 | 8.69 |
| #107 | B63 | 0.28 | 2.00 | 0.11 | 0.06 | 7.24 |
| #108 | B64 | 0.38 | 3.00 | 0.10 | 0.05 | 7.92 |
| #109 | B65 | 0.68 | 4.00 | 0.08 | 0.13 | 5.85 |
| #110 | B66 | 0.76 | 5.00 | 0.07 | 0.11 | 6.60 |
| #111 | B67 | 0.44 | 4.00 | 0.05 | 0.07 | 9.14 |
| #112 | B68 | 0.42 | 4.00 | 0.05 | 0.07 | 9.42 |
| #113 | B69 | 0.82 | 7.00 | 0.07 | 0.06 | 8.57 |
| #114 | B610 | 0.61 | 4.00 | 0.08 | 0.10 | 6.57 |
| #115 | B611 | 0.44 | 4.00 | 0.07 | 0.06 | 9.11 |
| #116 | B612 | 0.25 | 3.00 | 0.07 | 0.02 | 11.85 |
| #117 | B613 | 0.24 | 2.00 | 0.09 | 0.07 | 8.24 |
| #118 | B614 | 0.87 | 7.00 | 0.07 | 0.07 | 8.00 |
| #119 | B71 | 0.32 | 2.00 | 0.06 | 0.20 | 6.17 |
| #120 | B72 | 0.26 | 2.00 | 0.07 | 0.13 | 7.58 |
| #121 | B73 | 0.25 | 2.00 | 0.10 | 0.06 | 7.86 |
| #122 | B74 | 0.27 | 2.00 | 0.08 | 0.12 | 7.52 |
| #123 | B75 | 0.23 | 2.00 | 0.09 | 0.05 | 8.64 |
| #124 | B76 | 0.32 | 2.00 | 0.10 | 0.11 | 6.22 |
| #125 | B81 | 0.23 | 2.00 | 0.10 | 0.03 | 8.68 |
| #126 | B82 | 0.32 | 3.00 | 0.07 | 0.05 | 9.30 |
| #127 | B83 | 0.46 | 4.00 | 0.08 | 0.05 | 8.68 |
| #128 | B91 | 0.24 | 2.00 | 0.10 | 0.03 | 8.30 |
| #129 | C11 | 0.05 | 4.00 | 0.00 | 0.01 | 83.76 |
| #130 | C12 | 0.06 | 3.00 | 0.00 | 0.02 | 52.73 |
| #131 | C13 | 0.13 | 3.00 | 0.00 | 0.06 | 22.95 |
| #132 | C14 | 0.55 | 8.00 | 0.00 | 0.07 | 14.64 |
| #133 | C21 | 0.10 | 3.00 | 0.00 | 0.05 | 28.69 |
| #134 | C22 | 0.11 | 3.00 | 0.00 | 0.05 | 28.04 |
| #135 | C23 | 1.09 | 4.00 | 0.00 | 0.36 | 3.67 |
| #136 | C31 | 0.14 | 6.00 | 0.00 | 0.02 | 41.47 |
| #137 | C32 | 0.28 | 11.00 | 0.01 | 0.02 | 39.23 |
| #138 | C41 | 0.31 | 6.00 | 0.01 | 0.05 | 19.30 |
| #139 | C51 | 0.73 | 17.00 | 0.00 | 0.04 | 23.33 |
|  |  | **Acoustic parameters** | | | | |
| **Sound number (#)** | **Code** | **Duration (s)** | **Pulse number** | **Pulse duration (s)** | **Interpulse duration (s)** | **Pulse rate** |
| #140 | C61 | 1.80 | 14.00 | 0.00 | 0.13 | 7.76 |
| #141 | C71 | 0.29 | 6.00 | 0.00 | 0.05 | 20.72 |
| #142 | C81 | 0.57 | 11.00 | 0.01 | 0.05 | 19.41 |
| #143 | C91 | 0.48 | 12.00 | 0.00 | 0.04 | 25.12 |
| #144 | C92 | 0.60 | 10.00 | 0.00 | 0.06 | 16.54 |
| #145 | C93 | 3.31 | 39.00 | 0.00 | 0.08 | 11.78 |
| #146 | C94 | 0.41 | 19.00 | 0.01 | 0.02 | 46.11 |
| #147 | C95 | 0.87 | 35.00 | 0.01 | 0.02 | 40.33 |
| #148 | C96 | 0.42 | 18.00 | 0.00 | 0.02 | 42.50 |
| #149 | D11 | 2.24 | 12.00 | 0.00 | 0.20 | 5.35 |
| #150 | D12 | 0.97 | 39.00 | 0.01 | 0.02 | 40.35 |
| #151 | D13 | 1.84 | 51.00 | 0.01 | 0.03 | 27.78 |
| #152 | D14 | 4.01 | 64.00 | 0.01 | 0.06 | 15.97 |
| #153 | D15 | 0.86 | 40.00 | 0.00 | 0.02 | 46.68 |
| #154 | D16 | 4.27 | 121.00 | 0.00 | 0.03 | 28.36 |
| #155 | D21 | 0.26 | 16.00 | 0.00 | 0.01 | 61.58 |
| #156 | D22 | 1.43 | 24.00 | 0.01 | 0.06 | 16.84 |
| #157 | D23 | 0.87 | 18.00 | 0.00 | 0.05 | 20.59 |
| #158 | D24 | 0.85 | 21.00 | 0.00 | 0.04 | 24.68 |
| #159 | D25 | 0.87 | 47.00 | 0.01 | 0.01 | 53.83 |
| #160 | D26 | 0.81 | 20.00 | 0.01 | 0.04 | 24.75 |
| #161 | D27 | 0.85 | 26.00 | 0.00 | 0.03 | 30.44 |
| #162 | D28 | 0.32 | 3.00 | 0.00 | 0.15 | 9.47 |
| #163 | D29 | 0.07 | 3.00 | 0.00 | 0.03 | 45.15 |
| #164 | D210 | 1.82 | 20.00 | 0.01 | 0.09 | 11.02 |
| #165 | D211 | 0.69 | 39.00 | 0.00 | 0.01 | 56.27 |
| #166 | D212 | 0.81 | 9.00 | 0.00 | 0.10 | 11.10 |
| #167 | D31 | 0.19 | 5.00 | 0.00 | 0.04 | 25.87 |
| #168 | D32 | 0.39 | 4.00 | 0.00 | 0.13 | 10.29 |
| #169 | D33 | 0.55 | 5.00 | 0.00 | 0.13 | 9.07 |
| #170 | D34 | 0.46 | 5.00 | 0.00 | 0.11 | 10.76 |
| #171 | D35 | 0.81 | 9.00 | 0.01 | 0.10 | 11.11 |
| #172 | D41 | 0.51 | 6.00 | 0.00 | 0.10 | 11.83 |
| #173 | D42 | 0.93 | 5.00 | 0.01 | 0.22 | 5.39 |
| #174 | D43 | 0.05 | 4.00 | 0.00 | 0.01 | 79.83 |
| #175 | D51 | 0.09 | 6.00 | 0.00 | 0.01 | 69.35 |
| #176 | D52 | 0.57 | 5.00 | 0.01 | 0.13 | 8.83 |
| #177 | D53 | 0.43 | 6.00 | 0.00 | 0.08 | 13.96 |
| #178 | D54 | 0.09 | 5.00 | 0.00 | 0.02 | 57.68 |
| #179 | D55 | 0.10 | 4.00 | 0.00 | 0.03 | 38.98 |
| #180 | D56 | 0.05 | 3.00 | 0.00 | 0.02 | 59.62 |
| #181 | D57 | 0.15 | 14.00 | 0.01 | 0.00 | 95.54 |
| #182 | D58 | 0.43 | 8.00 | 0.00 | 0.06 | 18.56 |
| #183 | D61 | 0.18 | 3.00 | 0.01 | 0.08 | 17.11 |
| #184 | D71 | 0.18 | 3.00 | 0.01 | 0.08 | 17.11 |
| #185 | D81 | 0.49 | 5.00 | 0.01 | 0.11 | 10.12 |
| #186 | D82 | 0.30 | 26.00 | 0.00 | 0.01 | 85.36 |
